# Phenological optimization of late reproductive phase for raising wheat yield potential in irrigated mega-environments

**DOI:** 10.1093/jxb/erac144

**Published:** 2022-04-06

**Authors:** Pengcheng Hu, Scott C Chapman, Sivakumar Sukumaran, Matthew Reynolds, Bangyou Zheng

**Affiliations:** CSIRO Agriculture and Food, Queensland Biosciences Precinct, 306 Carmody Rd, St Lucia, Queensland 4067, Australia; The University of Queensland, School of Agriculture and Food Sciences, St Lucia, Queensland 4072, Australia; The University of Queensland, School of Agriculture and Food Sciences, St Lucia, Queensland 4072, Australia; International Maize and Wheat Improvement Centre (CIMMYT), Carretera México-Veracruz Km 45, El Batán, Texcoco, México, CP 56237, Mexico; International Maize and Wheat Improvement Centre (CIMMYT), Carretera México-Veracruz Km 45, El Batán, Texcoco, México, CP 56237, Mexico; CSIRO Agriculture and Food, Queensland Biosciences Precinct, 306 Carmody Rd, St Lucia, Queensland 4067, Australia; CONICET – National University of La Plata, Argentina

**Keywords:** Breeding, crop model, harvest index, mega-environment, phenology, spring wheat, yield potential

## Abstract

Increasing grain number through fine-tuning duration of the late reproductive phase (LRP; terminal spikelet to anthesis) without altering anthesis time has been proposed as a genetic strategy to increase yield potential (YP) of wheat. Here we conducted a modelling analysis to evaluate the potential of fine-tuning LRP in raising YP in irrigated mega-environments. Using the known optimal anthesis and sowing date of current elite benchmark genotypes, we applied a gene-based phenology model for long-term simulations of phenological stages and yield-related variables of all potential germplasm with the same duration to anthesis as the benchmark genotypes. These diverse genotypes had the same duration to anthesis but varying LRP duration. Lengthening LRP increased YP and harvest index by increasing grain number to some extent and an excessively long LRP reduced YP due to reduced time for canopy construction for high biomass production of pre-anthesis phase. The current elite genotypes could have their LRP extended for higher YP in most sites. Genotypes with a ratio of the duration of LRP to pre-anthesis phase of about 0.42 ensured high yields (≥95% of YP) with their optimal sowing and anthesis dates. Optimization of intermediate growth stages could be further evaluated in breeding programmes to improve YP.

## Teaser

Lengthening the late reproductive phase (LRP) of spring wheat increases yield potential and harvest index by increasing grain number in irrigated environments, but an excessively long LRP reduces yield potential.

## Introduction

Yield potential remains a principal breeding target as it is directly related to the actual on-farm production (Acreche *et al.*, 2008; Fischer and Edmeades, 2010). A substantial rise in grain yield potential is required to meet the continuously growing global demand for food security in the near future (Foulkes *et al.*, 2011; Reynolds *et al.*, 2011). The yield of wheat, a major grain food crop, is arguably limited by the grain sink strength during grain filling (Miralles and Slafer, 2007). Raising yield potential subsequently requires further improvement in the sink capacity through increasing grain number per unit land area and/or their average grain weight (Borrás *et al.*, 2004; Miralles and Slafer, 2007). Grain yield is largely determined by grain number, with grain weight having a much smaller effect on yield variations (Fischer, 1985), as grain growth is normally not limited by the availability of photosynthetic assimilated especially in high yielding genotypes in favourable environments (Borrás *et al.*, 2004; Fischer, 2007; Peltonen-Sainio *et al.*, 2007). The timing of phenological stages during the transition from vegetative growth through spike development to anthesis is anticipated to affect grain number and yield potential.

The phenological development in wheat from emergence to anthesis can be divided into vegetative phase (from emergence to floral initiation), early reproductive phase (from floral initiation to terminal spikelet initiation) and late reproductive phase (LRP; from terminal spikelet to anthesis) (Slafer and Rawson, 1994). The LRP coincides with the rapid growth of spikes and the development processes of florets while stem internodes elongate, which determine the survival of floret primordia to be fertile during anthesis and hence the final grain number (Kirby, 1988; Slafer and Rawson, 1994). The duration of LRP may affect grain number through the floret mortality and the possibility of floret primordia becoming fertile florets (Kirby, 1988; González *et al.*, 2003; Miralles and Slafer, 2007). A longer LRP allows (i) spikes to accumulate more assimilates for larger spike dry weight at anthesis, which is positively correlated with floret survival and fertility (Fischer, 1985; González *et al.*, 2011; Gonzalez-Navarro *et al.*, 2016), and/or (ii) floret primordia to have more time to develop and achieve the stage of fertile floret (Miralles *et al.*, 2000; Gaju *et al.*, 2009; Gonzalez-Navarro *et al.*, 2016). The duration of LRP is therefore a major determinant of yield potential (García *et al.*, 2011). Optimizing the phenological development pattern of pre-anthesis through tuning the onset of LRP (i.e. terminal spikelet) may contribute to increasing grain number and then rising wheat yield potential (Slafer *et al.*, 2001).

The duration of pre-anthesis phases (i.e. from emergence to anthesis) is determined by the sensitivity of the genotype to vernalization (cold temperature), photoperiod (day length), and average temperature (earliness *per se*) (Rousset *et al.*, 2011). The sensitivity to vernalization and photoperiod is mainly determined by the allele presence of vernalization and photoperiod genes, respectively. The allele presence of these genes enabled wheat genotypes to be classified as either a spring or winter type. Wheat plants (including completely ‘spring’ type) generally require a vernalization event to flower, followed by exposure to a lengthening photoperiod. The vernalization requirement of wheat is determined mainly by homoeologous *VRN1* genes, with the winter alleles of *VRN1* genes delaying anthesis time of spring type instead of stopping anthesis (Eagles *et al.*, 2009; Rousset *et al.*, 2011). The duration of each individual pre-anthesis phase may be partially independent of the others, as they seem to be under different genetic control and hence differ in sensitivity to vernalization, photoperiod, and temperature (Slafer and Rawson, 1994; Whitechurch *et al.*, 2007). For instance, Borràs-Gelonch *et al.* (2012) found that several quantitative trait loci (QTLs) had different effects on two pre-anthesis phases in two populations of spring lines, and several of these QTLs were significant for only one of the two phases. This relative independence among pre-anthesis phases makes it possible to fine-tune and optimize the duration of individual phases for raising yield potential (Halloran and Pennell, 1982; García *et al.*, 2011). It is important to emphasize that tuning the pre-anthesis phases should not change the timing of anthesis, as it is a major breeding objective of wheat adaptation to a target environment and avoids abiotic stresses (e.g. frost, heat, and drought) (Miralles and Slafer, 2007; Zheng *et al.*, 2012; Kamran *et al.*, 2014). The optimal anthesis period of a site is largely related to the location-specific environment (e.g. temperature, water, radiation, and frost and heat stresses) rather than genotype (Flohr *et al.*, 2017; Hunt *et al.*, 2019; Chen *et al.*, 2020; Hu *et al.*, 2021). The anthesis time of modern elite genotypes has been tuned to match the optimal anthesis period in most wheat production environments (Miralles and Slafer, 2007). For a given target environment (a combination of location, season, and sowing date), there may exist diverse genotypes that flower within the optimal anthesis period but vary in the phenological development patterns of the pre-anthesis phase and yield potential of wheat. A study on identifying the optimal development pattern of the wheat pre-anthesis phase for the target environment is particularly important for raising yield potential while minimizing the risk of abiotic stresses through gross changes in the timing of vegetative and reproductive development.

The selection of wheat genotypes with the optimal development patterns of pre-anthesis for the target environment could be conducted through field experiments across multiple growing seasons with diverse genotypes and sowing dates. Numerous studies supported the idea that modifying the development pattern of pre-anthesis resulted in changes in the number of fertile florets and then grain number under controlled or field conditions, through environmental manipulation of temperature and photoperiod and/or genetic manipulation of the sensitivity to these environmental factors (e.g. González *et al.*, 2003, 2005; Borràs-Gelonch *et al.*, 2012; Sanna *et al.*, 2014; Gonzalez-Navarro *et al.*, 2016; Basavaraddi *et al.*, 2021). However, these studies were mostly aimed at exploring the underlying physiological basis and limited to certain growing seasons, genotypes, and environments. To the best of our knowledge, rarely has a study focused on the identification of the genotypes with the optimal development patterns of pre-anthesis for further raising yield potential in target environments. Crop models could be used to augment empirical experiments to evaluate the potential of fine-tuning the development pattern of pre-anthesis in improving yield potentials of wheat through the simulation of genotype × environment × management interactions (Chenu *et al.*, 2017; Cooper *et al.*, 2021). Some crop models (e.g. APSIM-Wheat and DSSAT-Wheat) assume that more production of aboveground dry matter at anthesis leads to greater grain number without looking specifically at the spike growth and grain setting during LRP (Jones *et al.*, 2003; Holzworth *et al.*, 2014).

The objectives of this study were to use a gene-based phenology model (APSIM-Wheat-G; Zheng *et al.*, 2013) to (i) explore the response of yield-related variables (e.g. grain number and yield, aboveground biomass, and harvest index) to the varying duration of LRP without altering the anthesis date, and (ii) evaluate the potential of fine-tuning the development patterns of pre-anthesis in raising yield potentials at 70 representative sites of irrigated mega-environments (ME1 and ME5) for spring wheat.

## Materials and methods

### Simulation of phenological phases and grain yield in irrigated mega-environments

The International Maize and Wheat Improvement Center (CIMMYT) has used the concept of mega-environment (ME) to target germplasm development of wheat and distribute international nurseries for testing breeding lines regarding yield potential and adaptation to MEs. A ME is a subset of unnecessarily contiguous areas that share similar quantitative and geospatial criteria including climate and soil characteristics, cropping system requirements, and biotic and abiotic stresses (Rajaram *et al.*, 1995; Hodson and White, 2007). ME1 is defined as low rainfall, optimal irrigation, and highly productive spring wheat environments, and ME5 is characterized as warm and humid tropical or subtropical regions that may also need to be irrigated (see Supplementary Fig. S1; Hodson and White, 2007; Braun *et al.*, 2010). Spring wheat in these irrigated MEs is autumn-sown and about 50 million ha of wheat was grown in 2014 (Crespo-Herrera *et al.*, 2017).

A gene-based phenology module (Zheng *et al.*, 2013) integrated into the widely used crop model APSIM-Wheat (version 7.6; Holzworth *et al.*, 2014) was used to simulate the phenological stages and grain yield of sites in irrigated MEs. Briefly, the gene-based phenology module related the sensitivity to vernalization (*R*_v_) and photoperiod (*R*_p_) in APSIM-Wheat to the number of sensitive alleles of the *VRN1* (i.e. *Vrn-A1*, *Vrn-B1*, *Vrn-D1*) and *PPD1* (i.e. *Ppd-D1*) genes, with 0 for the spring or photoperiod insensitive alleles and 1 for the winter or photoperiod sensitive alleles. Linear functions were used to simulate the relationships between the weighted numbers of *VRN1* or *Ppd-D1* alleles (weighting and summing the values of 0 or 1 at each locus) and *R*_v_ or *R*_p_ (Zheng *et al.*, 2013). The integrated model (hereafter, APSIM-Wheat-G) requires inputs including daily weather data, management practices, soil characteristics, and genetic information for cultivars (i.e. alleles of *VRN1* and *Ppd-D1* genes). The gene information was used in the gene-based phenology module within the model to predict the wheat phenology. Compared with the default APSIM-Wheat, the APSIM-Wheat-G model has two modifications related to phenology modelling: (i) the interaction between vernalization and photoperiod effects was modelled by a multiplicative function (Weir *et al.*, 1984) instead of minimum function; and (ii) the photoperiod effect was extended to the anthesis stage as the photoperiod effects on pre-anthesis stages were widely reported (Slafer and Rawson, 1994). The APSIM-Wheat-G model has demonstrated its prediction performance for wheat phenology by combining genotypic and phenotypic data, which obtained a root mean square error (RMSE) value of 4.3 d for predicting 4475 observations of heading dates of 179 Australian genotypes at 79 sites across the Australian wheatbelt (Zheng *et al.*, 2013) and an RMSE of 5.5 d when validating with 1591 observations from 77 CIMMYT genotypes at 70 sites across irrigated MEs (Hu *et al.*, 2021). More information on the gene-based phenology model can be found in Zheng *et al.* (2013) and Hu *et al.* (2021).

Grain number in APSIM is estimated by a relationship between grain number and aboveground dry weight at the phenological stage of anthesis (Holzworth *et al.*, 2014; Zheng *et al.*, 2014). The value of aboveground dry weight is potentially influenced by the dynamics of canopy development interacting with phenological stages. Final grain yield through to maturity is then affected by the crop growth rate, potential grain size, and re-translocation of pre-anthesis biomass in stem and spike. In this study, the phenology parameters affect the value of aboveground dry weight at anthesis and grain number set, although there can be additional impacts on re-translocation, etc. That is to say, this study isolated the phenological dynamics while appreciating multiple other physiological mechanisms affecting the establishment of grain number and size.

**Fig. 1.**
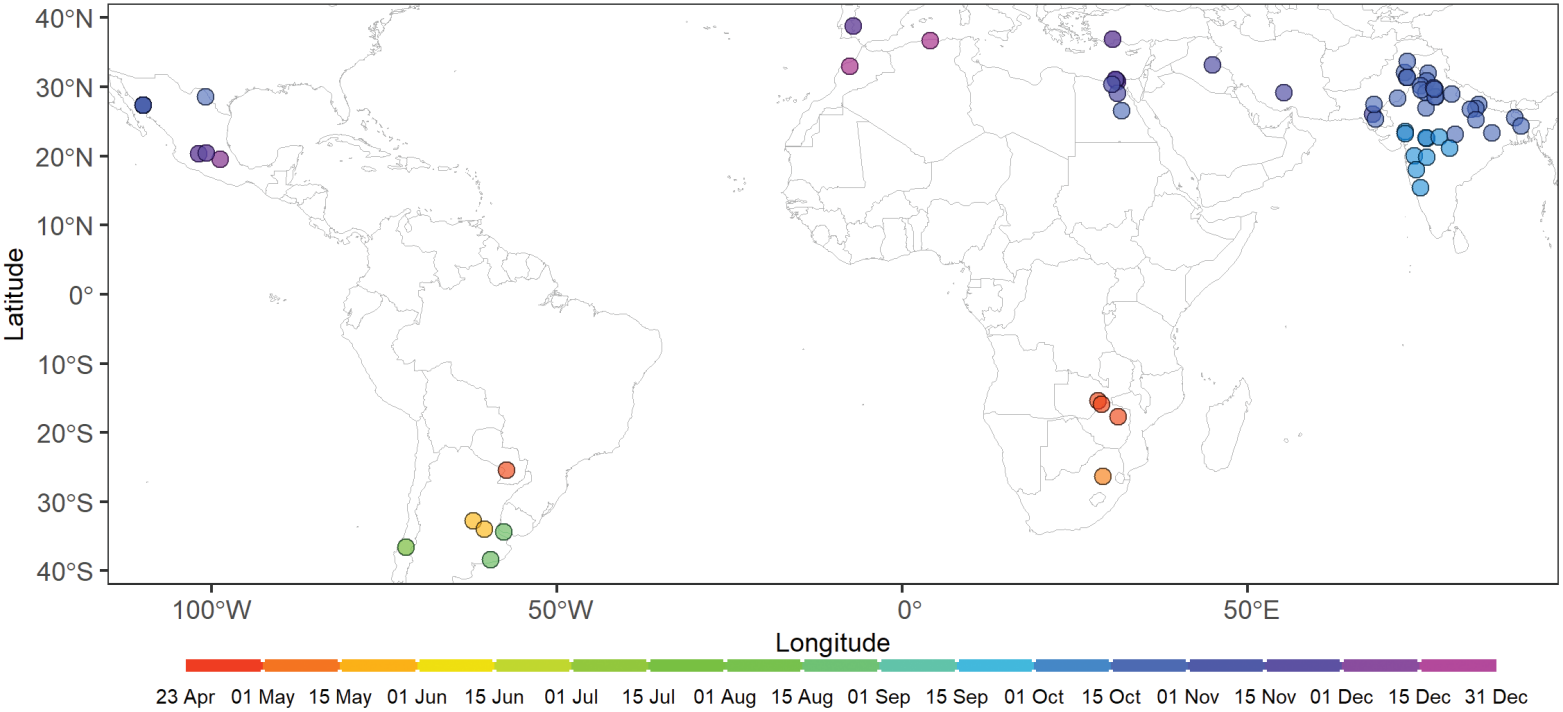
The geographical distribution of optimal sowing date of representative sites in irrigated mega environments. The optimal sowing date was required by the benchmark genotype in the 70 sites of irrigated mega-environments to flower at the optimal anthesis date (Supplementary Fig. S2) for the highest long-term mean yield (Hu *et al.*, 2021).

The APSIM-Wheat-G model was used to simulate phenological timing and grain yield of spring wheat using 34 years of climatic data (1985–2018) at the 70 sites in irrigated MEs (Fig. 1) as described by Hu *et al*. (2021). Briefly, these sites were used by the Wheat Yield Collaboration Yield Trial (WYCYT) and the Elite Spring Wheat Yield Trial (ESWYT), which were international multi-environment breeding nurseries that targeted the irrigated MEs (Sharma *et al.*, 2012; Sukumaran *et al.*, 2017). Daily weather data for these sites were derived from the gridded NASA POWER dataset with a spatial resolution of 0.5° (approximately 50 km, depending on latitude; Stackhouse *et al.*, 2011). The maximum distance to the centre of the closest grid cell was less than 18 km. The soil data were derived from the ISRIC WISE soil dataset (Batjes, 2012), which is a detailed geo-referenced global soil profile database with a spatial resolution of 5 arc-minute (approximately 10 km, depending on latitude) and was converted to compatible format with APSIM.

For each site, simulations were made for virtual genotypes and a real benchmark genotype of the site. The virtual genotypes were generated by the exhaustive combinations of cultivar-specific parameters controlling time to anthesis in APSIM-Wheat, including vernalization sensitivity (*R*_v_), photoperiod sensitivity (*R*_p_) and target thermal time from floral initiation to anthesis (TT_FI,FL_). Of these parameters, *R*_v_ and *R*_p_ ranged from 0 to 5 at 0.1 intervals and TT_FI,FL_ varied from 250 to 950 degree days (°Cd) at an interval of 25 °Cd. Subsequently, a total of 75 429 virtual genotypes were simulated at each site. The benchmark genotype of a given site was derived from our previous study (Hu *et al.*, 2021), which used a modelling approach (also with the APSIM-Wheat-G model) to identify the optimal anthesis and sowing date of the site with the benchmark genotype, considering the effects of frost and heat stresses on yield. The benchmark genotype corresponded to the highest long-term (1985–2018) mean yield of that site across different combinations of sowing dates (31 sowing dates within a 5-month sowing window) and parameterized genotypes (77 elite genotypes) from the WYCYT and ESWYT. More information on the parameterization of these genotypes with phenological and genetic data and modelling analysis of optimal anthesis and sowing dates at 70 sites of irrigated MEs can be found in Hu *et al.* (2021).

The simulated wheat crop was sown at the optimal sowing date of the site (Fig. 1) at a depth of 50 mm with a plant density of 250 plants m^−2^. The optimal sowing date could ensure the benchmark genotype of the site flowers at the optimal anthesis date (see Supplementary Fig. S2) for the highest long-term mean yield while minimizing the frost and heat stresses. Irrigation of 15 mm was applied at the sowing date to ensure emergence after sowing. The wheat crop was simulated with no water and nitrogen limitations, as (i) ME1 and ME5 are environments where wheat grows under near-full irrigation or sufficient rainfall, such that water is never a limiting factor (Hodson and White, 2007); (ii) water and nitrogen deficits were rarely observed in these trials as they were well irrigated and fertilized according to local management practices; and (iii) this study focused on evaluating the potential of raising yield potential by tuning phenological development patterns of pre-anthesis.

### Evaluation of the effects of tuning the late reproductive phase on yield formation

Three phenological stages were outputted from each APSIM-Wheat-G simulation, including emergence (DC10; Zadoks *et al.*, 1974), terminal spikelet (DC31), and anthesis (DC65). Five variables related to yield formation were also outputted, two of which are determined at anthesis (i.e. aboveground dry weight at anthesis (AGDW_AN_) and grain number (GN)), and the other three are determined at maturity (i.e. grain yield (GY), aboveground dry weight at physiological maturity (AGDW_PM_), and harvest index (HI)). The grain weight was excluded as the preliminary results of this study showed that grain weight remained constant with varying duration of LRP across sites, which was consistent with the literature suggesting that grain weight is conservative as grain growth was normally not limited by the availability of photosynthetic assimilates during the grain filling phase in diverse environments and genotypes (Fischer, 1985, 2007; Borrás *et al.*, 2004; Peltonen-Sainio *et al.*, 2007).

To evaluate the effects of fine-tuning LRP on yield potential, for each site we selected genotypes that had the same long-term (1985–2018) mean anthesis date (i.e. the same duration of pre-anthesis) as the benchmark genotype, with a difference of no more than 0.5 d. The duration of a phenological phase was expressed as thermal time and calculated from key growth stages of APSIM outputs. Daily thermal time (ΔTT_*i*_) was calculated with the daily minimum (*T*_min_) and maximum (*T*_max_) temperatures from climate records and three cardinal temperatures of wheat, i.e. 0 °C (base), 26 °C (optimum), and 34 °C (maximum) as in APSIM-Wheat (Eqs (1) and (2)) (Zheng *et al.*, 2014). All the genotypes were assumed to have the same cardinal temperatures in this study while acknowledging that the cardinal temperatures may vary with genotypes and phenological stages in wheat (Slafer and Rawson, 1995; Porter and Gawith, 1999). The duration of a phenological phase (TT_p_; Eq. (3)) was calculated as the summation of daily thermal time within the phase (*p*). The ratio of the duration of LRP (i.e. from terminal spikelet initiation to anthesis; TT_TA_) to the pre-anthesis phase (i.e. from emergence to anthesis; TT_EA_) was calculated. Hereafter, the ratio was referred to as *R*_TA/EA_ (Eq. (4)).


$$\begin{array}{l} T_{u}=\;\;\;\left(\frac{T_{min}+T_{max}}{2}\right)-\;T_{base} \end{array}$$ (1)

$$\begin{array}{l} \Delta TT_{i}=\left\{ \begin{array}{ll} T_{u}, & 0<T_{u}<26\\ \frac{26}{8}\left(34-T_{u}\right), & 26<T_{u}\leq 34\\ 0, & T_{u}\leq 0\;or\;T_{u}>34 \end{array}\right. \end{array}$$ (2)

$$\begin{array}{l} TT_{p}=\;\underset{i=1}{\overset{n}{\sum }}\;\Delta TT_{i} \end{array}$$ (3)

$$\begin{array}{l} R_{TA/EA}=\;\;\;\frac{TT_{TA}}{TT_{EA}} \end{array}$$ (4)

*T*_u_ is the thermal unit, *T*_base_ is the base temperature (°C), *i* is the *i*-th day within LRP (*p*=TA) or the pre-anthesis phase (*p=*EA), and *n* is the number of days within a phase.

To quantify the effects of the variation in TT_TA_ on these variables related to yield formation, the variability of AGDW_AN_, GN, AGDW_PM_, GY, and HI across the virtual genotypes with the same duration of pre-anthesis was calculated using the coefficient of variation (CV, %) at each site. To quantify the relationships between *R*_TA/EA_ and the five variables of the virtual genotypes, Spearman’s correlation (*r*) was calculated at a significance level of 0.05 (i.e. α=0.05) for each site. The simulated long-term mean GY of a genotype was considered as its yield potential as the wheat crop was simulated with no water and nitrogen limitations and flowered at the optimal anthesis date of the site to minimize the frost and heat stress (Hu *et al.*, 2021). The virtual genotype achieving the highest yield potential (GY_ph_) was selected for each site and the optimal TT_TA_ and then *R*_TA/EA_ obtained. GY_ph_ was compared with the simulated yield potential of the benchmark genotype (GY_pb_) of the given site to quantify the percentage increase in GY (*P*_GY_, %; Eq. (5)) for quantifying the effect of tuning TT_TA_ on raising yield potential. The optimal range of *R*_TA/EA_ was estimated for each site and defined as the *R*_TA/EA_ of genotypes that had the simulated long-term mean GY ≥95% of the GY_ph_ (i.e. 5% of yield loss) of that site. The optimal ranges of *R*_TA/EA_ were used to estimate a ratio ensuring high yields across sites in irrigated MEs. In addition, the daily average temperature and average cumulative radiation of the pre-anthesis phase (Supplementary Fig. S1) were calculated to explore their relationships with the CVs of these variables and *R*_TA/EA_ corresponding to GY_ph_. All the statistical analyses were implemented using customized R (R Core Team, 2019) scripts.


$$\begin{array}{l} P_{GY}=\;\;\;\frac{GY_{ph}-GY_{pb}}{GY_{pb}}\;\times \;100\;\%\; \end{array}$$ (5)

## Results

### Variability for variables of genotypes with the same duration to anthesis

**Fig. 2.**
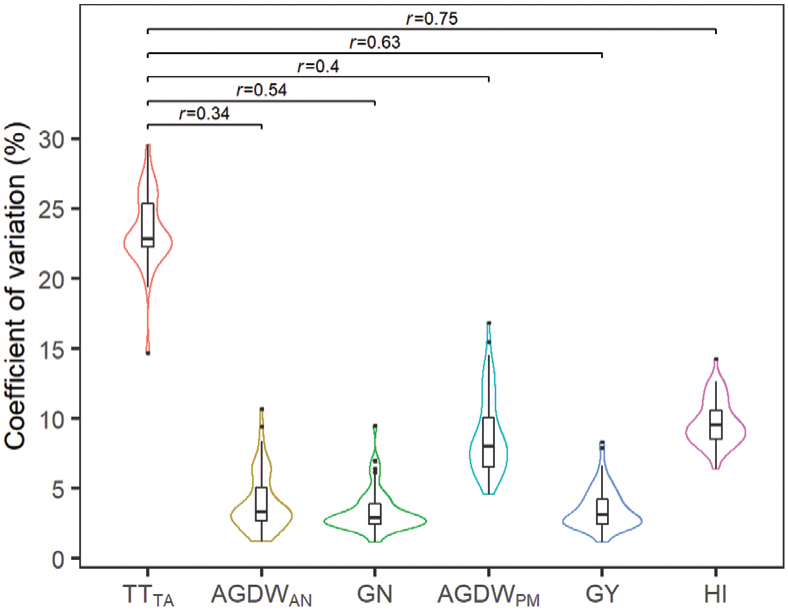
Distribution of coefficient of variation (%) of the duration of LRP (TT_TA_), aboveground dry weight at anthesis (AGDW_AN_), grain number (GN), aboveground dry weight at physiological maturity (AGDW_PM_), grain yield (GY), and harvest index (HI) across 70 sites in irrigated mega-environments. The coefficient of variation was calculated among virtual genotypes with the same duration to anthesis for each site. The violin plots represent the distributions of coefficients of variation of variables. The Spearman correlations (*r*) between the coefficients of variation of TT_TA_ and other variables are shown at the top of the figure.

**Fig. 3.**
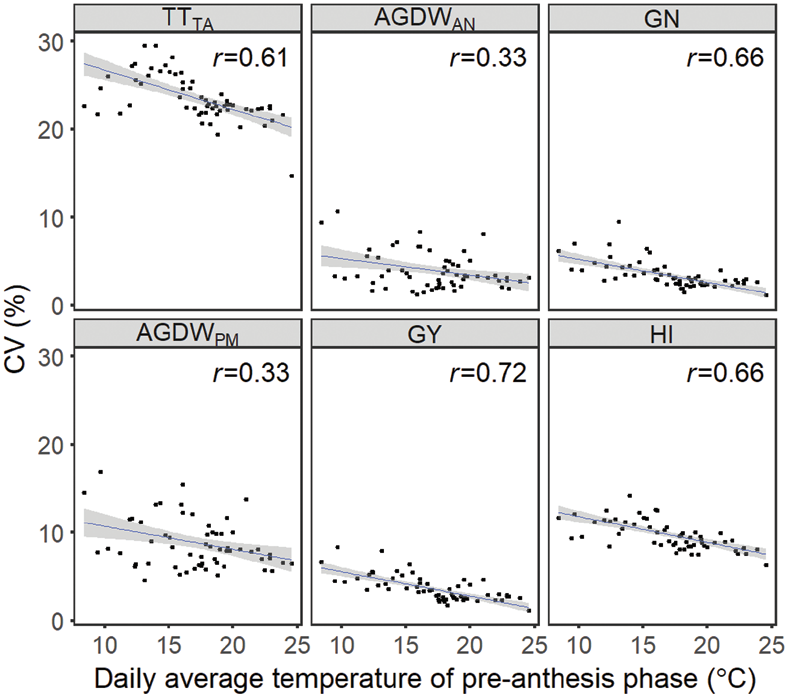
Relationship between the daily average temperature of pre-anthesis phase of benchmark genotypes and coefficients of variation (CV) of the duration of the late reproductive phase (TT_TA_), aboveground dry weight at anthesis (AGDW_AN_), grain number (GN), aboveground dry weight at physiological maturity (AGDW_PM_), grain yield (GY), and harvest index (HI) of virtual genotypes of spring wheat with the same duration to anthesis at sites of irrigated mega-environments.

The number of virtual genotypes with the same duration to anthesis varied from 96 to 1541 across sites, with most sites having 100–250 genotypes meeting this criterion (see Supplementary Fig. S3), depending on the intervals of parameter values used in this study. The simulated TT_TA_, AGDW_AN_, GN, AGDW_PM_, GY, and HI varied across virtual genotypes with the same duration to anthesis at each site (Fig. 2; Supplementary Fig. S4). For variables determined at anthesis, TT_TA_ showed the highest variability across sites, its CV ranging from 18.4% to 29.6% with a median of 22.8%. Both AGDW_AN_ and GN showed lower variability, having a similar range of less than 10% with a median of about 3%. For variables determined at maturity, AGDW_PM_ variability across sites ranged from 4.6% to 16.8% with a median of 8.0%. Variability of GY was from 1.2% to 8.3% and its mean value was 3.1%. Variability of HI was in the range of 7.5% to 14.2% with a mean value of 9.5%. Sites with a higher daily average temperature of the pre-anthesis phase tended to obtain lower variabilities of these variables (Fig. 3), with the average temperature closely correlated to variabilities of TT_TA_, GN, GY, and HI (all *r*>0.6) but with less effect on AGDW_AN_ and AGDW_PM_ (*r*=0.33). Subsequently, ME5 sites tended to have lower variabilities of these variables than ME1 sites (Supplementary Figs S5, S6). The TT_TA_ variability among sites was correlated with the variability of other variables (induced by the varying TT_TA_). Variability of TT_TA_ showed strong effects on GN, GY, and HI variability (*r*>0.5). Variability of AGDW_AN_ and AGDW_PM_ was only partially explained by the TT_TA_ variability (*r*<0.4).

### Responses of variables to the varying duration of the late reproductive phase

**Fig. 4.**
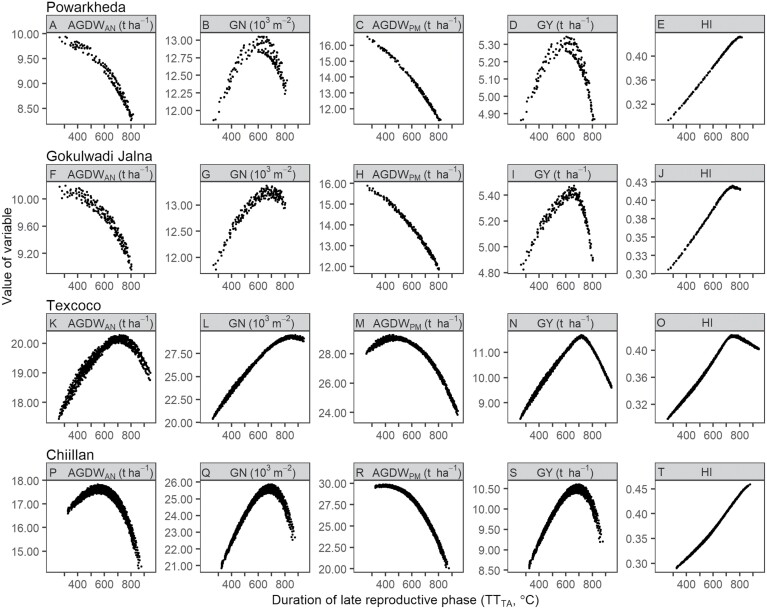
Examples of the relationships of simulated long-term (1985–2018) mean aboveground dry weight at anthesis (AGDW_AN_), grain number (GN), aboveground dry weight at maturity (AGDW_PM_), grain yield (GY), and harvest index (HI) against the duration of the late reproductive phase of virtual genotypes with the same duration to anthesis at sites of irrigated mega-environments. The four sites were located at Powarkheda (India), Gokulwadi Jalna (India), Texcoco (Mexico), and Chiillan (Chile).

The simulated AGDW_AN_, GN, AGDW_PM_, GY, and HI showed different responses to the varying TT_TA_ of virtual genotypes with the same duration to anthesis across sites (Fig. 4). These sites represent diverse response patterns of these variables to the varying TT_TA_ and different temperature and radiation conditions. The daily average temperatures of the pre-anthesis phase of these sites were 22.4 °C (Powarkheda), 22.8 °C (Gokulwadi Jalna), 13.1 °C (Texcoco), and 11.2 °C (Chiillan); the average cumulative radiations were 1293.3 MJ m^−2^ (Powarkheda), 1254.4 MJ m^−2^ (Gokulwadi Jalna), 2067.3 MJ m^−2^ (Texcoco), and 1912.4 MJ m^−2^ (Chiillan) (Supplementary Fig. S1). AGDW_AN_ and AGDW_PM_ had a similar response to the increased TT_TA_, i.e. they consistently decreased (as in Powarkheda and Gokulwasi Jalna; Fig. 4A, C, F, H) or initially increased to a maximum value and then decreased (as in Texcoco and Chiillan; Fig. 4K, M, P, R). Conversely, GN and HI increased at first (as in Powarkheda and Chiillan; Fig. 4E, T) or then decreased after increasing to the maximum value (as in Gokulwasi Jalna and Texcoco; Fig. 4G, J, L, O) when TT_TA_ incremented. GY first increased as TT_TA_ was extended until a maximum yield and then decreased when TT_TA_ was further increased (as in Powarkheda, Gokulwasi Jalna, Texcoco, and Chiillan; Fig. 4D, I, N, S). Although only four representative sites are demonstrated in Fig. 4, the other sites in this study showed similar patterns.

**Fig. 5.**
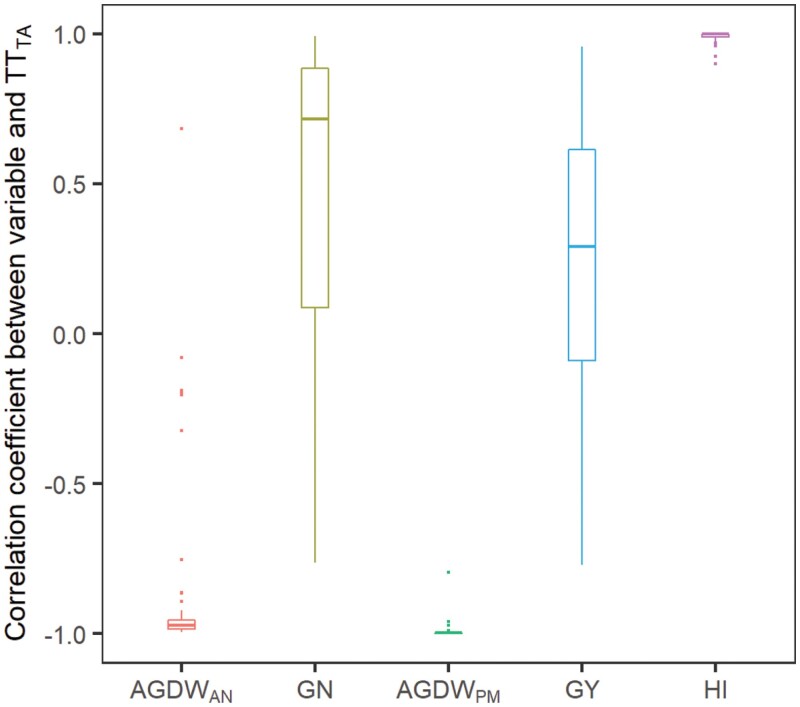
Variation of Spearman correlation between the duration of the late reproductive phase (TT_TA_) and aboveground dry weight at anthesis (AGDW_AN_), grain number (GN), aboveground dry weight at physiological maturity (AGDW_PM_), grain yield (GY), and harvest index (HI) across 70 sites in irrigated mega-environments. The Spearman correlation was calculated using genotypes with the same duration to anthesis for each site. Spatial distributions of the Spearman correlation between the duration of the late reproductive phase and these variables are presented in Supplementary Figs S7, S8.

Both AGDW_AN_ and AGDW_PM_ were closely and negatively correlated with TT_TA_ (Fig. 5; Supplementary Figs S7, S8) across most sites. The Spearman correlation coefficient (*r*) varied from −0.99 to 0.68 for AGDW_AN_, with most of them (63 out of 70 sites) less than −0.8, showing that AGDW_AN_ consistently decreased with TT_TA_ increased (e.g. Fig. 4A, F). Some sites with lower average temperatures (latitude >34° and/or high altitude) showed less correlation between AGDW_AN_ and TT_TA_ (−0.8<*r*<0.8), indicating that AGDW_AN_ initially increased to a maximum value and then decreased as TT_TA_ increased (e.g. Fig. 4K, P). AGDW_PM_ was highly and negatively correlated with TT_TA_ across sites (*r*<−0.8). Both GN and GY showed a large variation in correlations with TT_TA_ across sites, with *r* varying from −0.76 to 0.99 for GN and from −0.77 to 0.96 for GY. GN was positively and closely correlated with TT_TA_ (*r*>0.8) at 30 sites and so was GY at eight sites, indicating that they increased with TT_TA_ (e.g. Fig. 4B). GN and GY in the other sites followed the pattern of initially increasing to a maximum value and then decreasing as TT_TA_ increased (e.g. Fig. 4D, Q). HI was positively and closely correlated with TT_TA_, with *r* ranging from 0.90 to 0.99, showing that HI tended to monotonically increase along with increased TT_TA_ at most sites (e.g. Fig. 4E, T).

### Yield potential and harvest index raised by fine-tuning the late reproductive phase

**Fig. 6.**
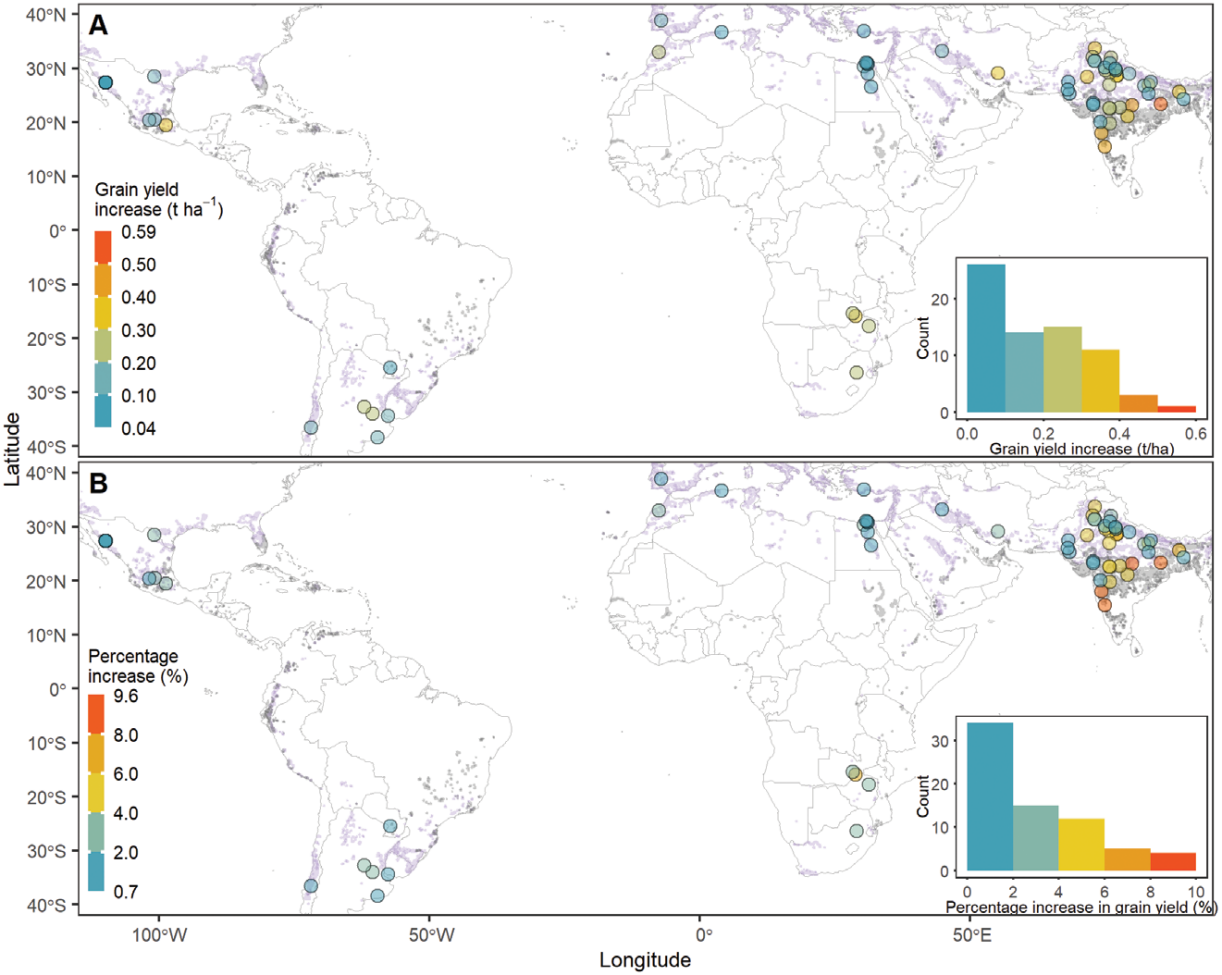
Amount (A) and percentage (B) of increase in simulated (1985–2018) yield potential at 70 sites of irrigated mega-environments. The increases in yield potential were calculated by the comparison of the virtual genotype with the optimal duration of the late reproductive phase and the benchmark genotype of the site. The two genotypes had the same duration to anthesis but different duration of the late reproductive phases.

Across years, the simulated mean GY_ph_ varied across sites, ranging from 3.6 to 11.7 t ha^−1^ (Supplementary Fig. S9A). ME1 sites had higher GY_ph_ than ME5 sites, varying from 5.1 to 11.7 t ha^−1^ with a mean of 6.9 t ha^−1^. ME5 sites achieved lower GY_ph_ ranging from 3.6 to 5.9 t ha^−1^ with a mean of 5.1 t ha^−1^. Sites with higher latitudes or altitudes had the highest GY_ph_ (>8 t ha^−1^). When compared with the simulated GY_pb_ of sites, the increase in yield potential ranged from 0.04 to 0.59 t ha^−1^ with a mean of 0.2 t ha^−1^ (Fig. 6A). The increases in yield potential of ME1 sites were slightly smaller than ME5 sites on average, with a mean of 0.19 t ha^−1^ for ME1 sites and 0.22 t ha^−1^ for ME5 sites. The percentage increase in the yield potential varied between 0.7% and 9.6% across sites (Fig. 6B). ME5 sites had a larger percentage of yield potential increases than ME1 sites, with an overall average of 3.0% increase for ME1 sites and 4.6% for ME5 sites.

**Fig. 7.**
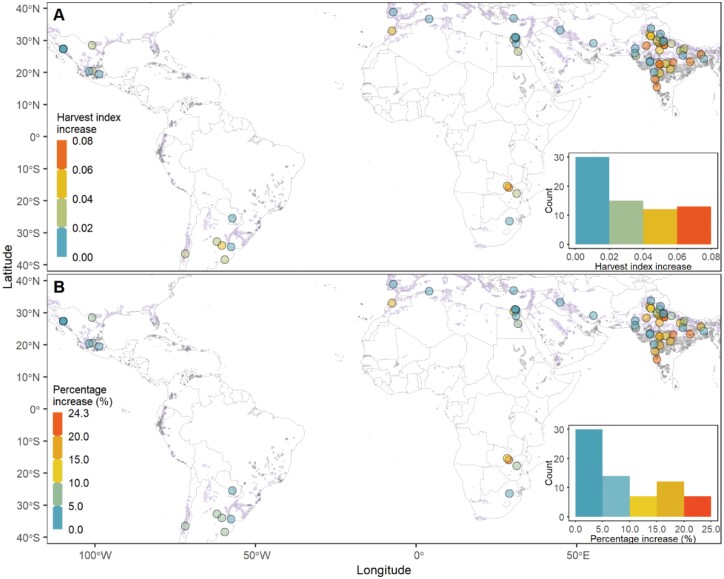
Amount (A) and percentage (B) of increase in simulated (1985–2018) harvest index corresponding to the highest yield potential of sites in irrigated mega-environments. The increases in harvest index were calculated by the comparison between the virtual genotype with the optimal duration of the late reproductive phase and the benchmark genotype of the site. The two genotypes had the same duration to anthesis but different durations of the late reproductive phases.

The simulated HI corresponding to the highest yield potential ranged from 0.34 to 0.43 with a mean of 0.40 (Supplementary Fig. S9B). ME1 sites had higher HI than ME5 sites, with HI of ME1 sites varying from 0.36 to 0.43 with a mean of 0.40 and ME5 sites ranging between 0.34 and 0.41 with a mean of 0.39. When compared with the HI of the benchmark genotype, the amount of increase in HI of the genotype with the highest yield potential ranged from 0 to 0.08 with a mean of 0.03 (Fig. 7A). The percentage of increase in the HI varied between 0 and 24.3% across sites with a mean of 8.9% (Fig. 7B). ME5 sites had a larger percentage HI increase than ME1 sites, with an overall average of 8.5% increase for ME1 sites and 10.8% for ME5 sites.

**Fig. 8.**
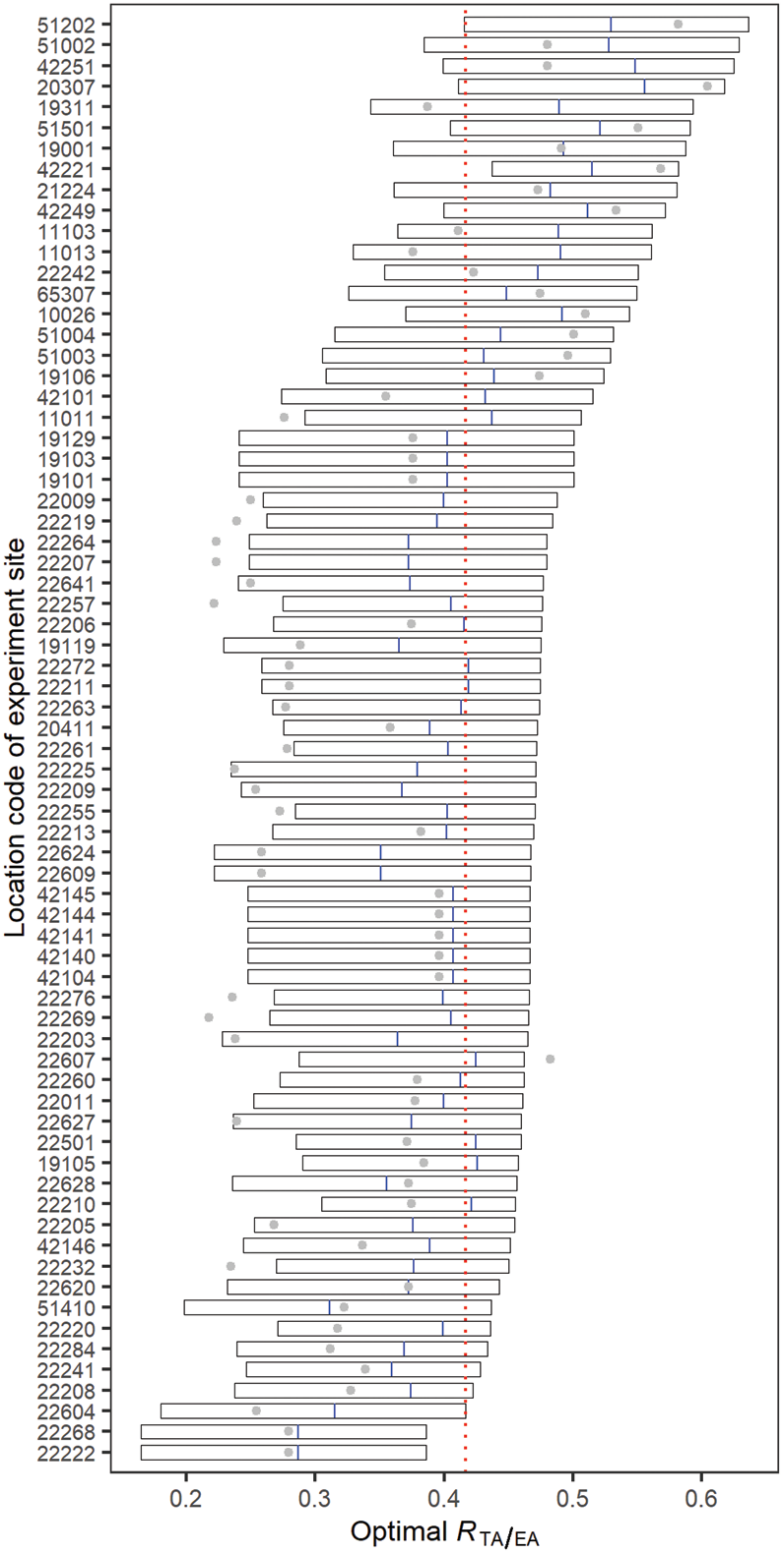
Optimal spans of the ratio of the duration of the late reproductive to pre-anthesis phases (*R*_TA/EA_) for the highest yields (≥95% of the highest yield potential) of 70 sites in irrigated mega-environments. Rectangles are the optimal ranges of the ratio of the highest grain yields of the sites. Blue segments are the *R*_TA/EA_ of the genotypes with the highest yield potentials and the grey circles are the *R*_TA/EA_ of the benchmark genotypes. The red dotted line is the *R*_TA/EA_ ensuring the highest yields across most sites (67 out of 70 sites).

The corresponding genotypes for producing the GY_ph_ of the sites had different development patterns of pre-anthesis (Supplementary Fig. S10). The duration of pre-anthesis varied from 1159.2 to 1731.6 °Cd across sites. The TT_TA_ for GY_ph_ ranged between 503.8 and 771.5 °Cd. Subsequently, *R*_TA/EA_ for the GY_ph_ ranged between 0.29 and 0.56 across sites with a mean of 0.42 (Supplementary Fig. S9C). The *R*_TA/EA_ appeared to be negatively associated with the average temperature (*r*=0.63) but positively correlated with the average cumulative radiation (*r*=0.84) of the pre-anthesis phase (Supplementary Fig. S11). The optimal range of *R*_TA/EA_ for the highest GYs (≥95% of the GY_ph_) varied across sites (Fig. 8). The lowest and highest *R*_TA/EA_ of individual sites were 0.17 and 0.64, respectively. The span of the optimal range of *R*_TA/EA_ varied between 0.13 and 0.21 across sites, and an *R*_TA/EA_ of 0.42 could ensure the highest yields at most sites (67 out of 70 sites). The *R*_TA/EA_ of benchmark genotype was within the optimal range of *R*_TA/EA_ at most sites (58 out of 70 sites). The difference of *R*_TA/EA_ between the GY_ph_ and the benchmark genotype of each site ranged from −0.07 to 0.19. The *R*_TA/EA_ of the benchmark genotype was normally less than that of the GY_ph_ (56 out of 70 sites). Consequently, the TT_TA_ of benchmark genotypes should be extended to approach a larger *R*_TA/EA_ at these sites for a higher yield potential (Supplementary Fig. S12).

## Discussion

Increasing grain number per square metre through fine-tuning phenological development pattern of the pre-anthesis phase without altering the anthesis time was considered as a breeding strategy to increase the yield potential of wheat (González *et al.*, 2005; Miralles and Slafer, 2007). In a previous paper (Hu *et al.*, 2021), it was shown that the gene-based model (APSIM-Wheat-G) could be used to predict wheat anthesis time across irrigated MEs. The model was then used to identify the optimal anthesis and sowing date of the current elite genotype with the highest long-term mean yield at individual sites, considering the potential impacts of frost and heat stress. Based on these results, this study used a modelling approach with APSIIM-Wheat-G to evaluate the potential of fine-tuning the duration of LRP for further raising the yield potential of spring wheat in irrigated MEs through increasing sink strength.

There were multiple virtual genotypes with the same duration to anthesis at each site (see Supplementary Fig. S3), which agreed with experimental studies (e.g. Whitechurch and Slafer, 2002; Gonzalez-Navarro *et al.*, 2016) and indicated that diverse phenological combinations of genotypes achieved the same timing to anthesis. This was because the duration of these phases may be partially independent of each other, as the sensitivity of each phase to vernalization, photoperiod, and temperature seems to be under different genetic controls (Slafer and Rawson, 1994; González *et al.*, 2003; Borràs-Gelonch *et al.*, 2012). The varying TT_TA_ of virtual genotypes with the same duration to anthesis induced variations in variables related to yield formation (i.e. AGDW_AN_, GN, AGDW_PM_, GY, and HI). Variabilities of these variables appeared to be negatively associated with the daily average temperature of pre-anthesis (Fig. 3), perhaps because the benchmark genotypes of sites with mild temperatures tended to have a longer duration to anthesis, which could favour more genotypes with the same duration to anthesis but distinct partitioning of the pre-anthesis phases. Variabilities of these variables (medians of the CV of less than 10%) were significantly less than the TT_TA_ variability (a median of 22.8%), and their correlation coefficients with the TT_TA_ variability were less than 0.75 (Fig. 2), which suggested that the TT_TA_ variability was partially converted to these variables.

The response to varying TT_TA_ differed in different variables (Figs 4, 5). AGDW_AN_ gradually decreased as the TT_TA_ increased at most sites (e.g. Fig. 4A and F), which was due to that longer LRP consequently shortened the vegetative and early reproductive phases (from emergence to terminal spikelet) under a given duration to anthesis. A shorter duration of vegetative and early reproductive phase produced a small canopy with lower maximum leaf area index (see Supplementary Fig. S13), thus reducing the amount of biomass production at anthesis (Borràs-Gelonch *et al.*, 2012; Gonzalez-Navarro *et al.*, 2016). AGDW_PM_ similarly responded to TT_TA_ as AGDW_AN_ (e.g. Fig. 4C, H) since genotypes shared the same grain filling process. Lengthening LRP is usually recognized as an avenue to increase GN and then GY (Miralles and Slafer, 2007). Our study confirmed this idea with GN increasing as the TT_TA_ lengthened (e.g. Fig. 4B) at some sites (30 out of 70 sites), which was because lengthening LRP allowed more assimilates to be diverted to the growing spikes during LRP for an increased spike dry weight at anthesis, which resulted in higher number of fertile florets and GN (Fischer, 1985; Slafer *et al.*, 1990; Miralles and Slafer, 2007; Gaju *et al.*, 2009). However, as the TT_TA_ further lengthened, GN experienced a decline after achieving a maximum value (e.g. Fig. 4Q) at other sites, and this means that lengthening LRP for higher GN failed to compensate for the biomass production of a small canopy caused by shortened vegetative phase. It was reported that a longer LRP was not always related to a higher spike dry weight and then higher GN, as it might hamper canopy growth and/or biomass partitioning to spike (Gonzalez-Navarro *et al.*, 2016) or there might be a trade-off between duration and the rate of spike growth (González *et al.*, 2005). This experimental result is apparently captured at least in part in the simulations presented here. Further, lengthening the LRP without altering the timing of anthesis requires advancing the onset of stem elongation. The early onset of stem elongation may increase the spring frost risk during the sensitive LRP, which deforms young spikes or reduces the tiller and spike number and then GN (Fletcher and Cullis, 1988; Martino and Abbate, 2019). GY was closely correlated to GN, so the response of GY to varying TT_TA_ was similar to that of GN, i.e. GY decreased after reaching a maximum as the TT_TA_ increased (Fig. 4D, S) at most sites (62 out of 70 sites). The strong correlation between GY and GN implied that there was no effect of LRP variations on individual grain weight in this study. This was in agreement with previous studies, which concluded that wheat has a high degree of homeostasis in final grain weight as grain growth is mostly sink-limited during the grain filling phase (i.e. the assimilate availability exceeds the demand of growing grains) under broad combinations of growing environments and cultivars (Fischer, 1985, 2007; Borrás *et al.*, 2004; Peltonen-Sainio *et al.*, 2007). HI tented to increase with TT_TA_, which was mainly attributed to decreasing AGDW_PM_ and then followed by increasing GY. This also showed that the trade-off between AGDW_PM_ and HI suggested that pursuing larger AGDW_PM_ or higher HI alone was not sufficient for GY gain (Aisawi *et al.*, 2015; Reynolds *et al.*, 2017).

Yield potential could be raised by selecting or breeding genotypes with optimal phenological development patterns of pre-anthesis across sites of irrigated MEs (Fig. 6). The simulated highest yield potentials on the ME1 and ME5 sites were 6.9 t ha^−1^ and 5.1 t ha^−1^ on average, respectively (see Supplementary Fig. S9A), which represented a 3.0% and 4.6% increase on average, respectively, compared with the yield potential of the simulated benchmark genotypes of the sites. It should be noted that the yield potential was the further improvements on that of the benchmark genotype of the site, which was bred to raise the yield potential or selected from the current elite lines of spring wheat in irrigated environments (Trethowan *et al.*, 2003; Sharma *et al.*, 2012; Sukumaran *et al.*, 2017). The benchmark genotype was well adapted to the given environment for realizing its yield potential with the known optimal sowing and anthesis date of the site, which was evaluated by a comprehensive modelling analysis of genotype, environment, and management in the previous paper (Hu *et al.*, 2021). Therefore, it was expected that the relatively low percentage increase in yield potential (about 4% on average) was estimated in this study by fine-tuning LRP when compared with the current high-yielding genotype (i.e. the benchmark genotype). On the other hand, the simulated yield potential of the benchmark genotype was normally within the range of ≥95% of the highest yield potential of the site (58 out of 70 sites; Fig. 8). The HI corresponding to the highest yield potential increased by 8.9% on average across sites as compared with that of the benchmark genotypes (Fig. 7), which combined with the improved yield potential implied that the genotype with optimal duration of LRP might have an appropriate balance between source and sink and succeed in more fully exploiting assimilation capacity (Reynolds *et al.*, 2009).

The differences of *R*_TA/EA_ between these virtual genotypes with the highest yield potentials and corresponding benchmark genotypes suggested the strategy of fine-tuning LRP for further raising yield potential at individual sites (Fig. 8). The results showed that the duration of LRP of spring wheat (represented by the benchmark genotype) should be extended without altering anthesis time for high yield potential at most sites (56 out of 70 sites; see Supplementary Fig. S12). This agrees with experimental studies, which concluded that extending the LRP of wheat could raise yield potential by increasing GN without offsetting grain weight (Miralles and Slafer, 2007). The virtual genotypes with the highest yield potentials of the sites had varying *R*_TA/EA_ ranging from 0.29 to 0.56 (Supplementary Fig. S9C), but an *R*_TA/EA_ of about 0.42 could realize at least 95% of the highest yield potential (with a risk of 5% yield loss) across most sites (67 out of 70 sites; Fig. 8) with their optimal sowing and anthesis dates. The *R*_TA/EA_ of about 0.42 agreed with that of genotypes/cultivars of spring wheat under field conditions, which normally varied between 0.30 and 0.45 (Slafer *et al.*, 2001; Whitechurch *et al.*, 2007; Borràs-Gelonch *et al.*, 2012; Sanna *et al.*, 2014; Guo and Schnurbusch, 2015; Ochagavía *et al.*, 2017). Whitechurch *et al.* (2007) evaluated the variability in the duration of LRP of 64 Argentine wheat cultivars, and their *R*_TA/EA_ varied from about 0.27 to 0.39 when sown at the recommended sowing date, which was smaller than the *R*_TA/EA_ (≥0.43) of the highest yield potentials at the three Argentina sites (Supplementary Fig. S9C). Further, Whitechurch *et al.* (2007) reanalysed the 20 wheat cultivars reported in previous literature, whose *R*_TA/EA_ mostly ranged from about 0.27 to 0.51. Guo and Schnurbusch (2015) measured the TT_EA_ and TT_TA_ of 12 German spring cultivars and the *R*_TA/EA_ varied between 0.37 and 0.42 with a mean of 0.40. Variability in *R*_TA/EA_ was observed in populations of recombinant lines of wheat as well. Borràs-Gelonch *et al.* (2012) reported TT_EA_ and TT_TA_ of 212 recombinant lines of Australian spring cultivars and the calculated *R*_TA/EA_ ranged between about 0.33 and 0.47. Similarly, another population of 100 recombinant lines had *R*_TA/EA_ of about 0.22 to 0.41 with a mean of 0.34 under the long day and non-vernalized condition (Sanna *et al.*, 2014). The range of optimal *R*_TA/EA_ for high yields (≥95% of the highest yield potential) was an indicator of difficulty in the selection of the well-adapted genotypes for the site (Fig. 8), with a wide range suggesting more potential candidates for the target environment and vice versa.

Further work is required in terms of model development and analysis. This study focused on the potential of fine-tuning LRP in improving the sink strength and yield potential via boosting grain number, but altering the duration of the LRP may also change the potential grain weight (Calderini *et al.*, 1999; Calderini and Reynolds, 2000; Ugarte *et al.*, 2007). Thus potential grain weight will be considered in future modelling analysis of fine-tuning LRP for increasing yield potential. The terminal spikelet stage was not explicitly simulated by the current ASPIM-Wheat model; mechanistic models with a molecular and/or physiological basis to predict the terminal spikelet stage were developed (e.g. Brown *et al.*, 2013), but more effort is required to improving their validity. Further, crop processes of reproductive growth and development (e.g. floret fertilization, grain setting, and grain growth) need to be more specifically modelled to improve predictive performance (Brisson *et al.*, 2002; Messina *et al.*, 2019). Particularly, the effects of plant nitrogen status at anthesis on grain setting should be modelled as it was strongly correlated to wheat GN (Abbate *et al.*, 1995; Jeuffroy and Bouchard, 1999; Lemaire and Ciampitti, 2020). Nitrogen deficiency before anthesis could reduce GN, as it is related to the reductions in various components of grain set (e.g. the number of spikes per square metre, spikelets per spike, and differentiated florets, floret survival, and fertility) (Peltonen-Sainio and Peltonen, 1995; Demotes-Mainard *et al.*, 1999; Demotes-Mainard and Jeuffroy, 2001). In addition, the effects of post-anthesis nitrogen status on grain weight also should be considered in modelling grain yield formation and grain quality (Kimball *et al.*, 2001; Nuttall *et al.*, 2017; Zhao *et al.*, 2020).

### Conclusions

This study used a modelling approach to evaluate the potential of fine-tuning LRP of spring wheat in raising yield potential at sites of irrigated MEs. The aim was to isolate the phenological dynamics while appreciating that there are multiple other physiological mechanisms affecting the establishment of grain number and size. The simulation analysis demonstrated that diverse genotypes with the same duration to anthesis could vary in duration of LRP at individual sites. Lengthening LRP to some extent could increase the yield potential and harvest index of wheat by increasing grain number, with genotypes of the optimal duration of LRP achieving further increase (about 4% on average and up to 10%) in yield potential and harvest index (about 9% on average and up to 24%) compared with those of the high-yielding benchmark genotypes of the respective sites. Genotypes with a ratio of the duration of LRP to the pre-anthesis phase of about 0.42 could ensure at least 95% of the highest yield potential across most sites with their optimal sowing and anthesis dates. The current elite genotypes could have their LRP extended for higher yield potential in most sites. The results also implied that an excessively long duration of LRP reduced yield potential due to a reduction in time for canopy construction (with sufficient leaves and tillers) for high biomass production in the pre-anthesis stage. The study suggested that fine-tuning pre-anthesis development patterns without altering anthesis time can raise wheat yield potential by improving grain set, and therefore post-anthesis sink strength and HI.

## Supplementary data

The following supplementary data are available at *JXB* online.

Fig. S1. The long-term (1985–2018) daily average temperature and average cumulative radiation of the pre-anthesis phase of benchmark genotypes at 70 sites in irrigated mega-environments.

Fig. S2. The geographical distribution of optimal flowering date of the 70 representative sites in irrigated mega-environments.

Fig. S3. The distribution of the number of virtual genotypes with the same duration to anthesis at 70 sites of irrigated mega-environments.

Fig. S4. Variations in the duration of the late reproductive phase, aboveground dry weight at anthesis and grain number, aboveground dry weight at physiological maturity, grain yield, and harvest index of virtual genotypes of spring wheat with the same duration to anthesis at 70 sites of irrigated meta-environments.

Fig. S5. Coefficient of variation (%) in the duration of the late reproductive phase, aboveground dry weight at anthesis and grain number of virtual genotypes of spring wheat with the same duration to anthesis at 70 sites of irrigated mega-environments.

Fig. S6. Coefficient of variation (%) in the aboveground dry weight at physiological maturity, harvest index and grain yield of virtual genotypes of spring wheat with the same duration to anthesis at 70 sites of irrigated mega-environments.

Fig. S7. Spearman correlation between the duration of late reproductive phase and two variables determined at anthesis, aboveground biomass and grain number, of virtual genotypes of spring wheat with the same duration to anthesis at 70 sites of irrigated mega-environments.

Fig. S8. Spearman correlation between the duration of late reproductive phase and three variables determined at maturity, aboveground biomass, harvest index, and grain yield, of virtual genotypes of spring wheat with the same duration to anthesis at 70 sites of irrigated mega-environments.

Fig. S9. Spatial distribution of the simulated (1985–2018) highest yield potentials, the corresponding harvest index, and the ratio of the duration of the late reproductive phase to pre-anthesis phase of genotype with the optimal duration of the late reproductive phase across sites of irrigated mega-environments.

Fig. S10. Durations of different pre-anthesis phases (°Cd) of the virtual genotypes with the highest yield potentials of spring wheat at 70 sites of irrigated mega-environments.

Fig. S11. Relationship between the ratio of the duration of the late reproductive phase to the pre-anthesis phase (*R*_TA/EA_) of the genotypes with highest yield potentials and their daily average temperature and average cumulative radiation of the pre-anthesis phase of sites in irrigated mega-environments.

Fig. S12. Strategies for fine-tuning the duration of the late reproductive phase of the benchmark genotypes to approach the highest yield potential at 70 sites in irrigated mega-environments.

Fig. S13. Spearman correlation between the duration of late reproductive phase and the maximum leaf area index of virtual genotypes of spring wheat with the same duration to anthesis at 70 sites of irrigated mega-environments.

erac144_suppl_Supplementary_Figures_S1-S13

## Data Availability

The data supporting the findings of this study are available from the corresponding author (BZ) upon request.

## Abbreviations

AGDW_AN_: aboveground dry weight at anthesis
AGDW_PM_: aboveground dry weight at physiological maturity
GN: grain number
GY: grain yield
HI: harvest index
LRP: late reproductive phase
ME: mega-environment
TT_TA_: duration of the late reproductive phase (from terminal spikelet to anthesis) expressed in thermal time
YP: yield potential
