# Supplementary material for: Phenological optimization of late reproductive phase for raising wheat yield potential in irrigated mega-environments
Source: J Exp Bot. 2022 Apr 6;73(12):4236–49. doi: 10.1093/jxb/erac144 (PMC9232205; doi:10.1093/jxb/erac144)
Supplement: erac144_suppl_Supplementary_Figures_S1-S13 [file erac144_suppl_supplementary_figures_s1-s13.pdf]

Supplementary

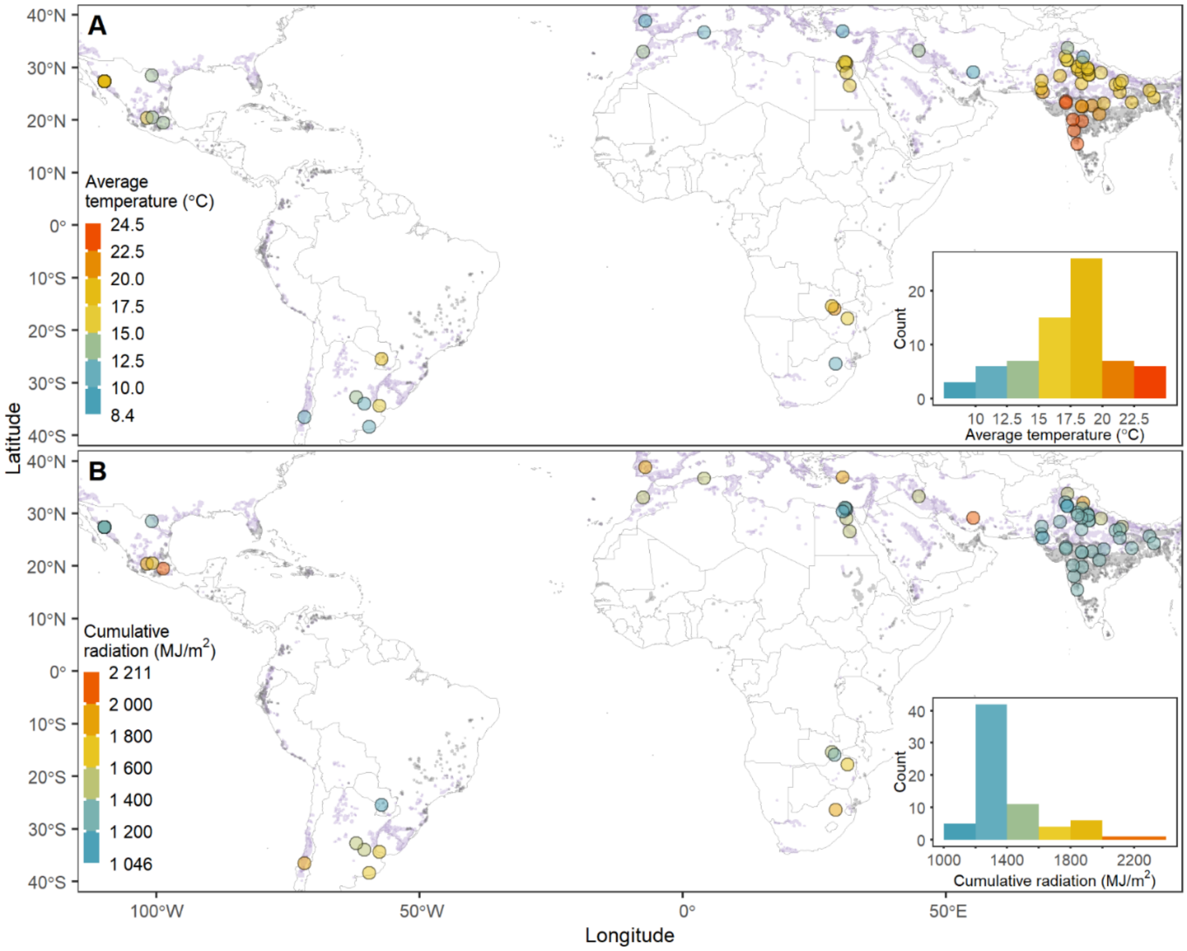

Fig. S1 The long-term (1985-2018) daily average temperature (A) and average cumulative radiation of the pre-anthesis phase of benchmark genotypes at 70 sites in irrigated mega-environments (MEs). The benchmark genotypes were sown at the optimal sowing dates to flower at the optimal flowering dates of sites. Purple and grey shadings denote ME1 and ME5, respectively.

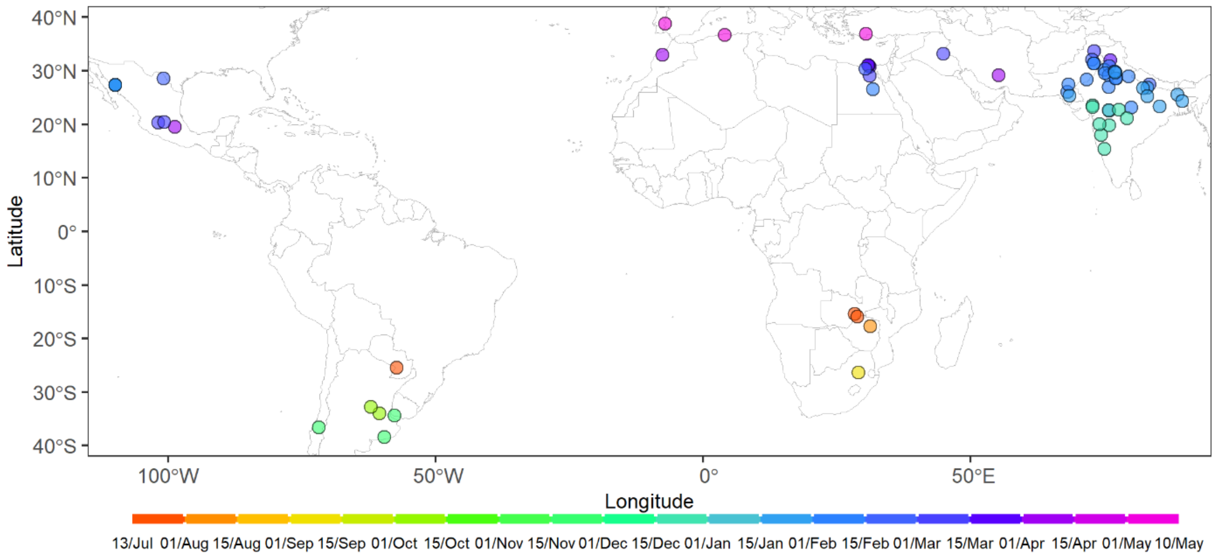

Fig. S2 The geographical distribution of optimal flowering date of the 70 representative sites in irrigated mega-environments.

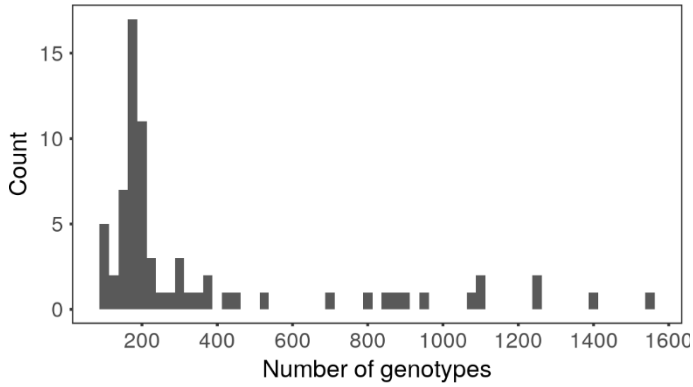

Fig. S3 The distribution of the number of virtual genotypes with the same duration to anthesis at 70 sites of irrigated mega-environments.

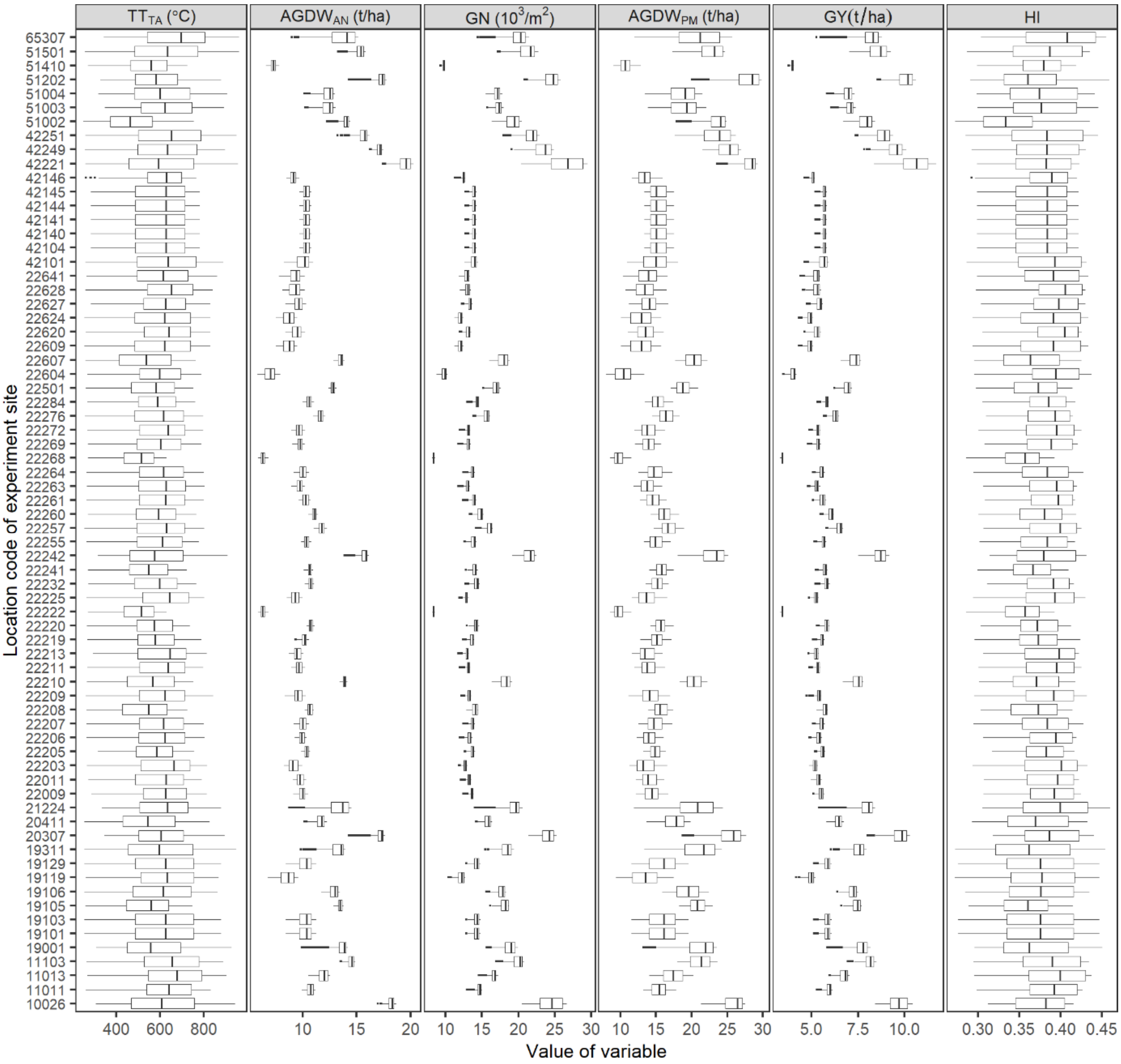

Fig. S4 Variations in the duration of the late reproductive phase (TT_{TA}), aboveground dry weight at anthesis ($AGDW_{AN}$) and grain number (GN), aboveground dry weight at physiological maturity ($AGDW_{PM}$), grain yield (GY) and harvest index (HI) of virtual genotypes of spring wheat with the same duration to anthesis at 70 sites of irrigated meta-environments.

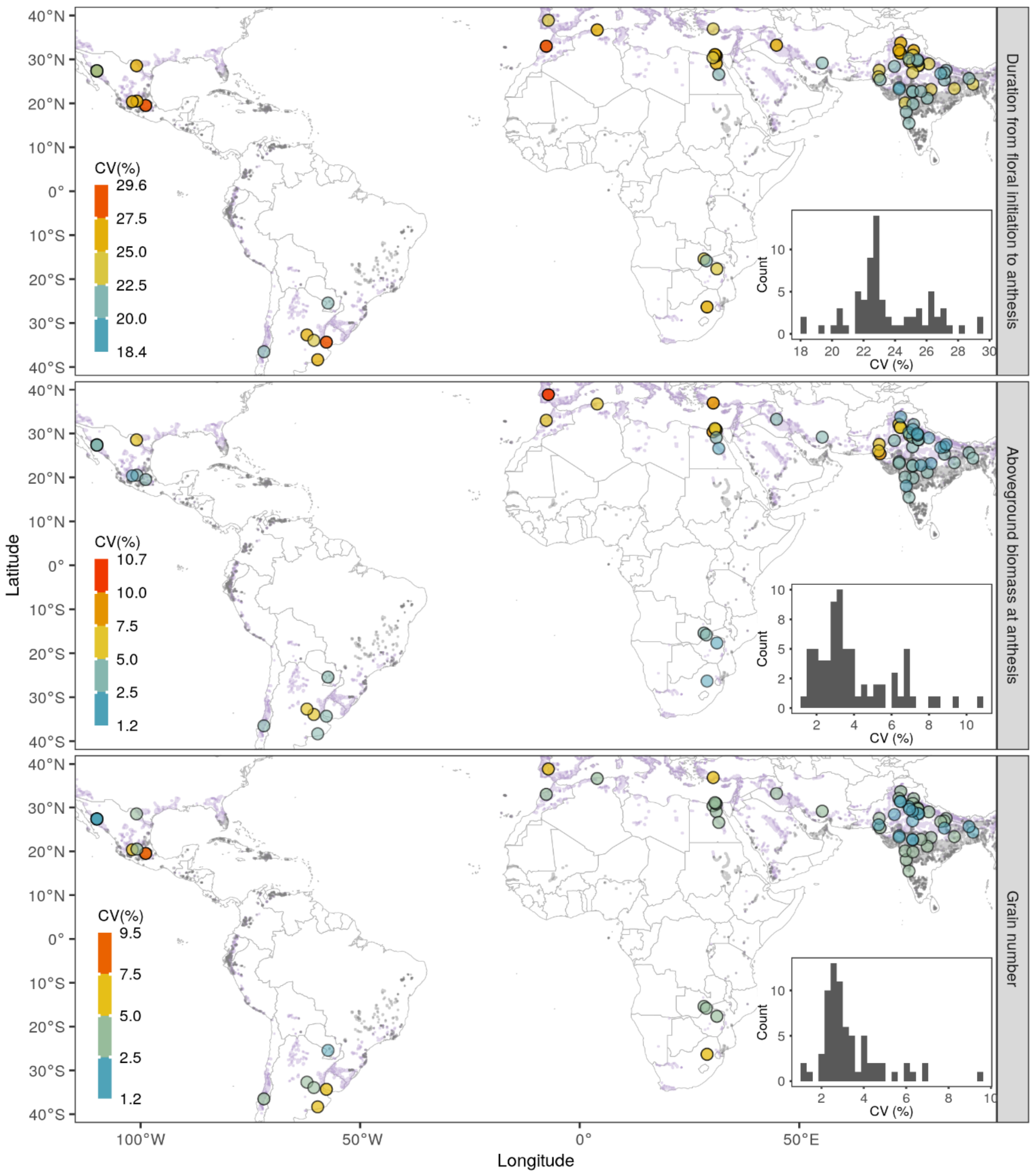

Fig. S5 Coefficient of variation (%) in the duration of the late reproductive phase, aboveground dry weight at anthesis and grain number of virtual genotypes of spring wheat with the same duration to anthesis at 70 sites of irrigated mega-environments.

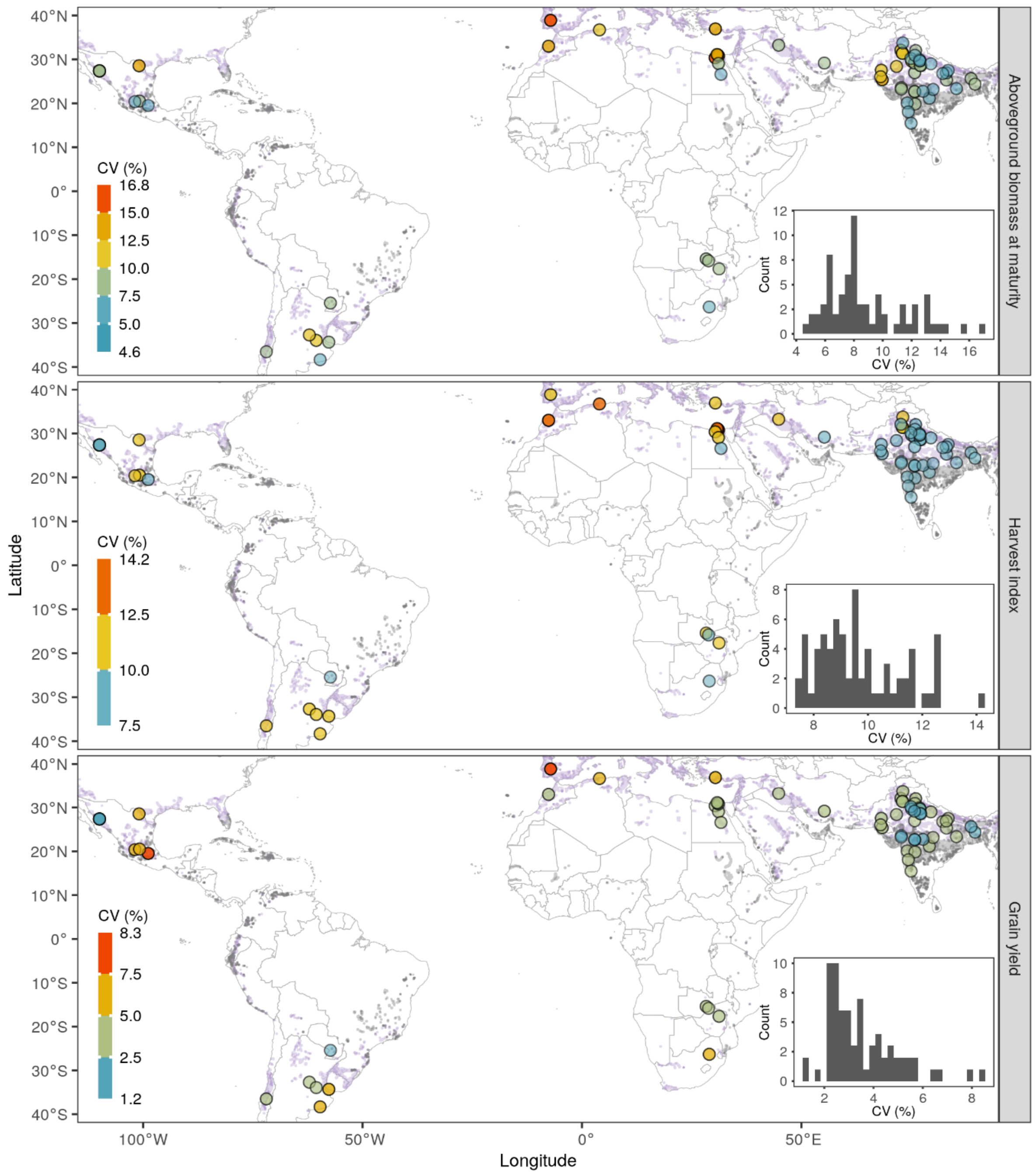

Fig. S6 Coefficient of variation (%) in the aboveground dry weight at physiological maturity, harvest index and grain yield of virtual genotypes of spring wheat with the same duration to anthesis at 70 sites of irrigated mega-environments.

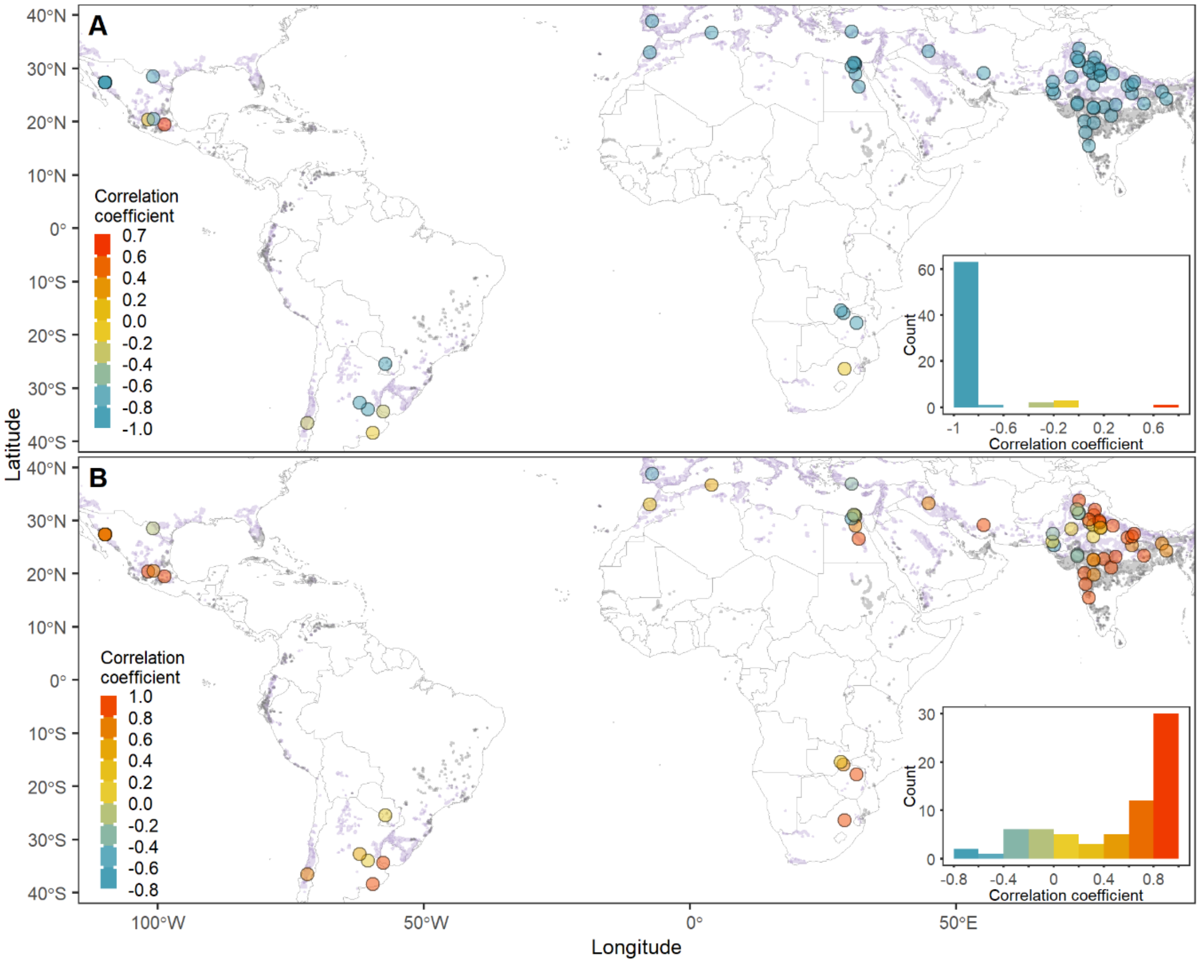

Fig. S7 Spearman correlation between the duration of late reproductive phase and two variables determined at anthesis: aboveground biomass (A) and grain number (B) of virtual genotypes of spring wheat with the same duration to anthesis at 70 sites of irrigated mega-environments.

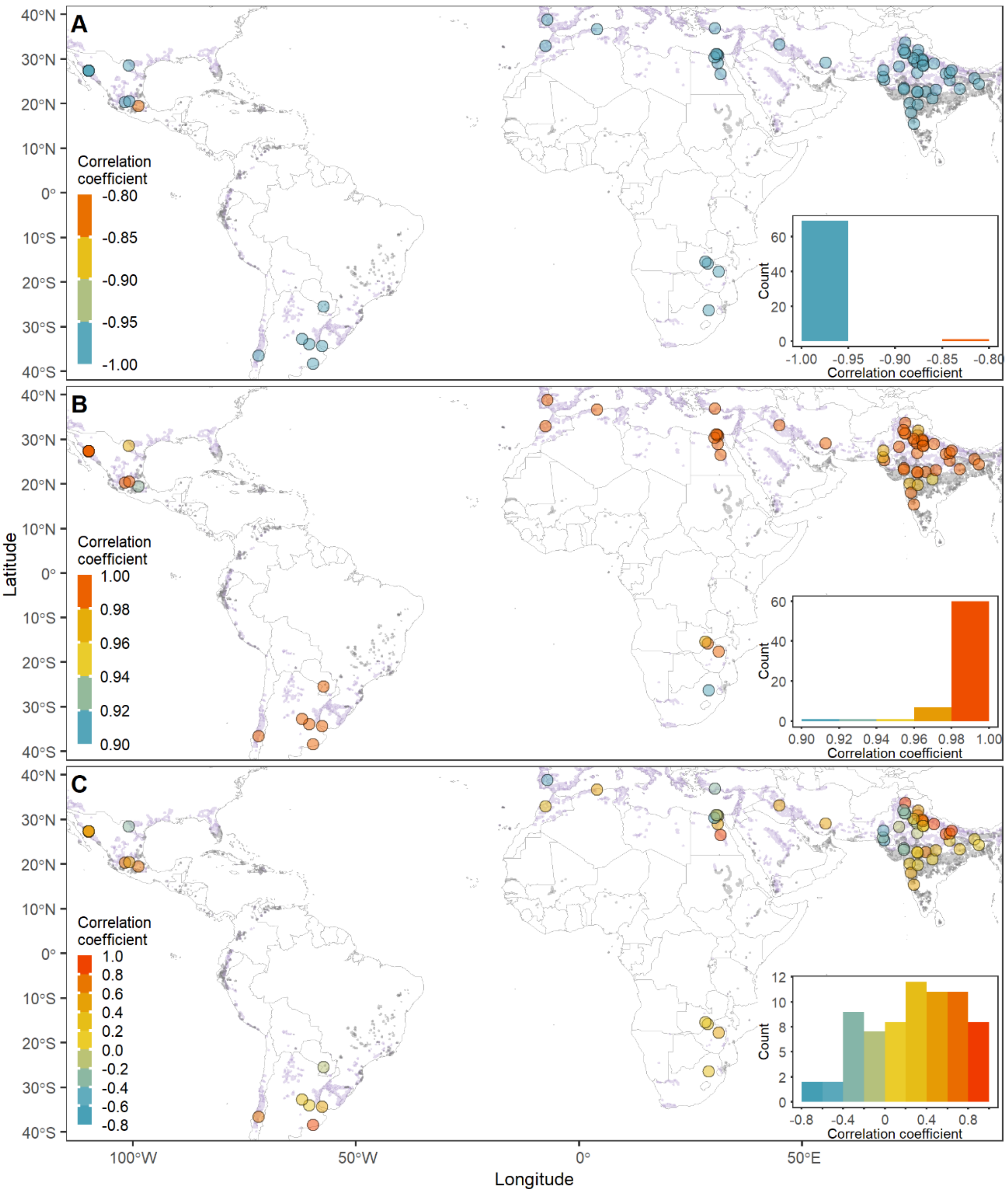

Fig. S8 Spearman correlation between the duration of late reproductive phase and three variables determined at maturity: aboveground biomass (A), harvest index (B) and grain yield (C) of virtual genotypes of spring wheat with the same duration to anthesis at 70 sites of irrigated mega-environments.

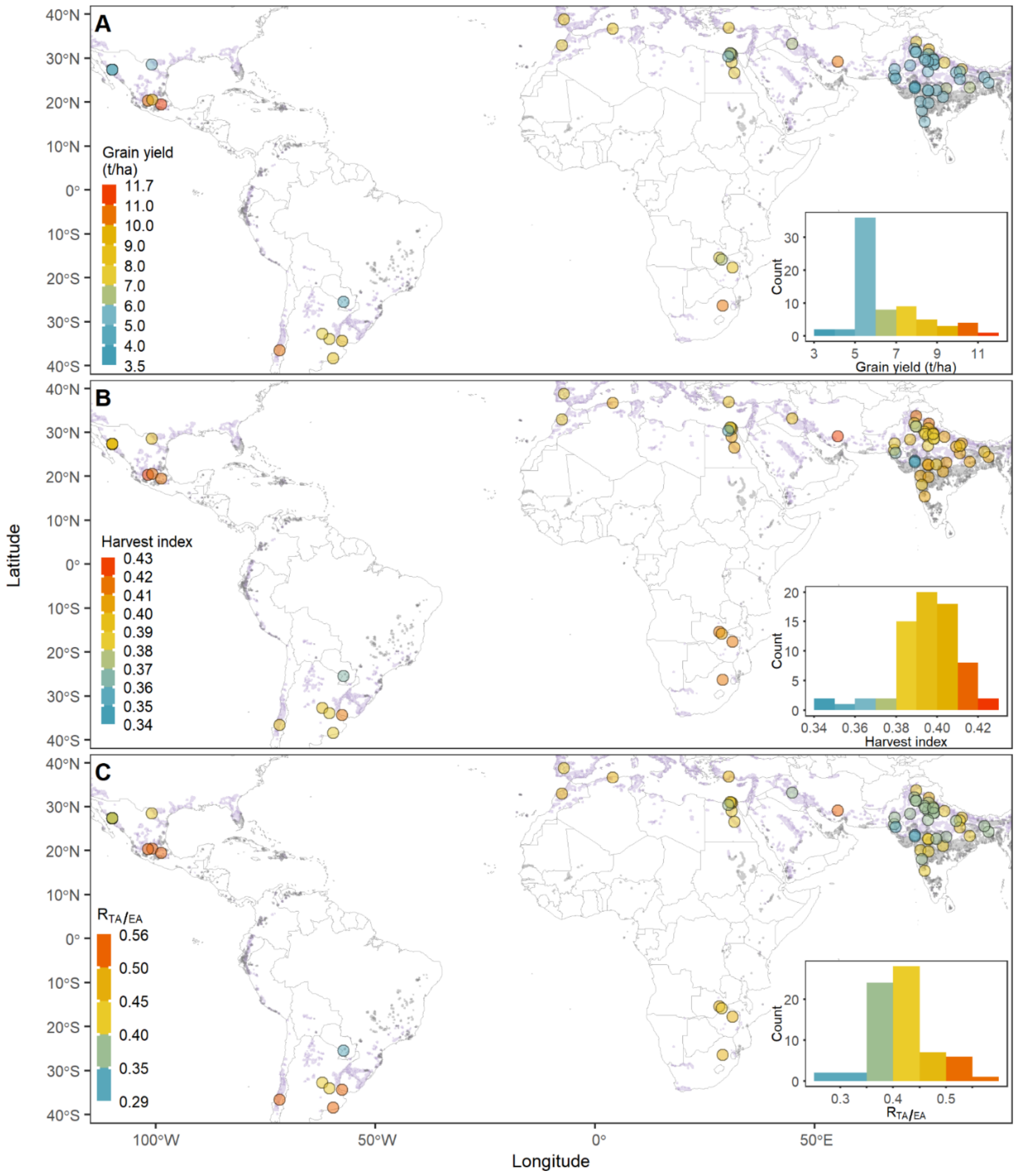

Fig. S9 Spatial distribution of the simulated (1985-2018) highest yield potentials (A), the corresponding harvest index (B) and the ratio of the duration of the late reproductive phase to pre-anthesis phase (C) of genotype with the optimal duration of the late reproductive phase across sites of irrigated mega-environments.

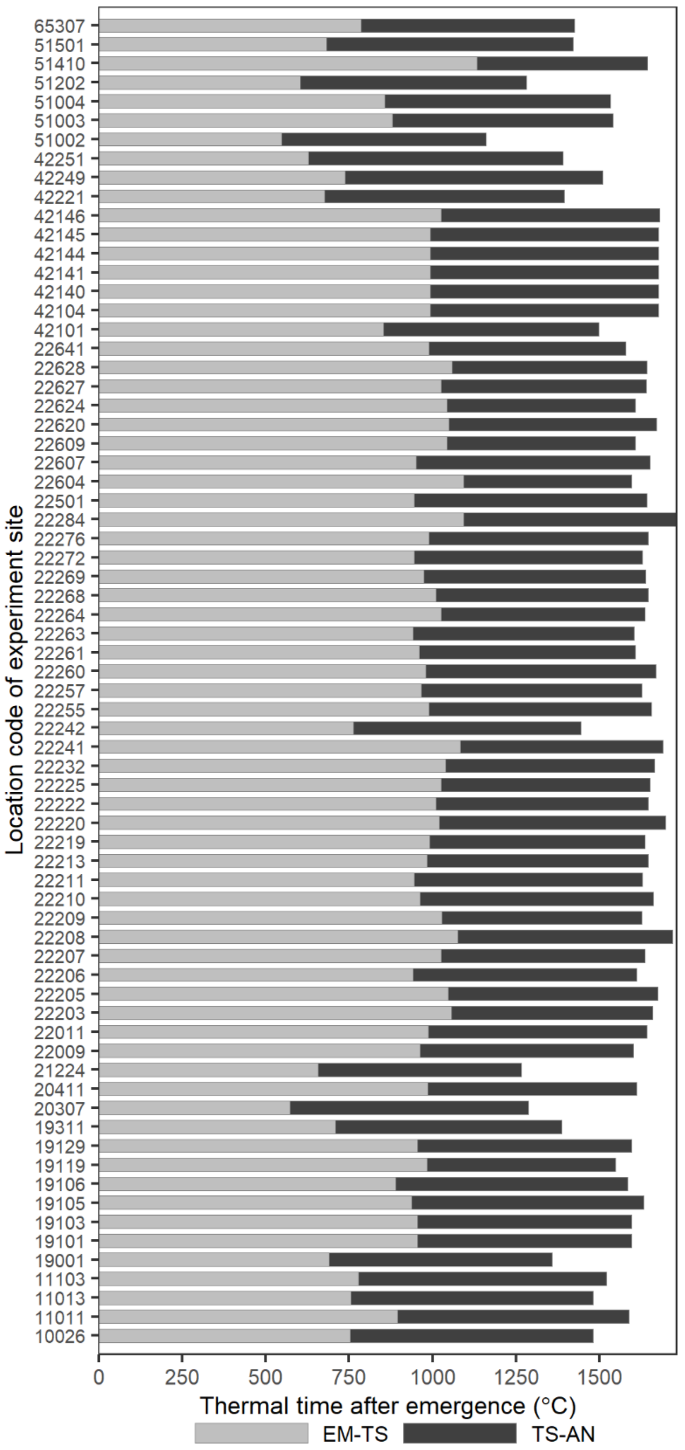

Fig. S10 Durations of different pre-anthesis phases (°Cd) of the virtual genotypes with the highest yield potentials of spring wheat at 70 sites of irrigated mega-environments. The rectangle dashed with light and dark grey are the duration from emergence to terminal spikelet initiation (EM-TS) and the late reproductive phase (from terminal spikelet initiation to anthesis (TS-AN)), respectively.

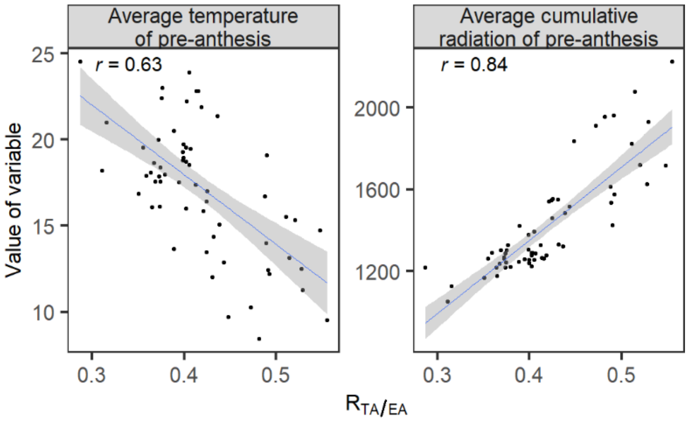

Fig. S11 Relationship between the ratio of the duration of the late reproductive phase to the pre-anthesis phase ($R_{TA/EA}$) of the genotypes with highest yield potentials and their daily average temperature (left) and average cumulative radiation (right) of the pre-anthesis phase of sites in irrigated mega-environments.

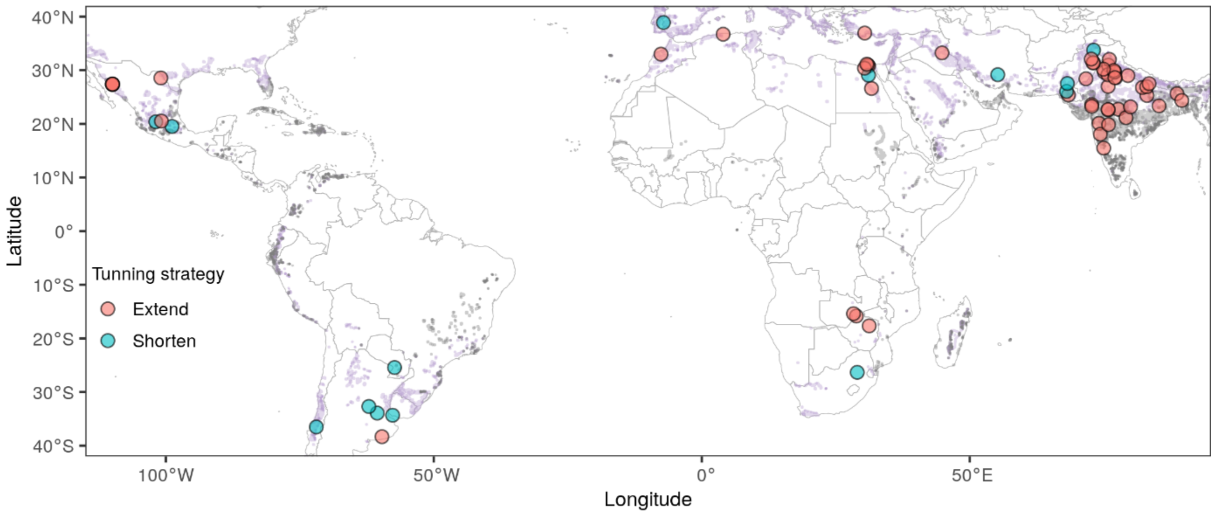

Fig. S12 Strategies for fine-tuning the duration of the late reproductive phase of the benchmark genotypes to approach the highest yield potential at 70 sites in irrigated mega-environments.

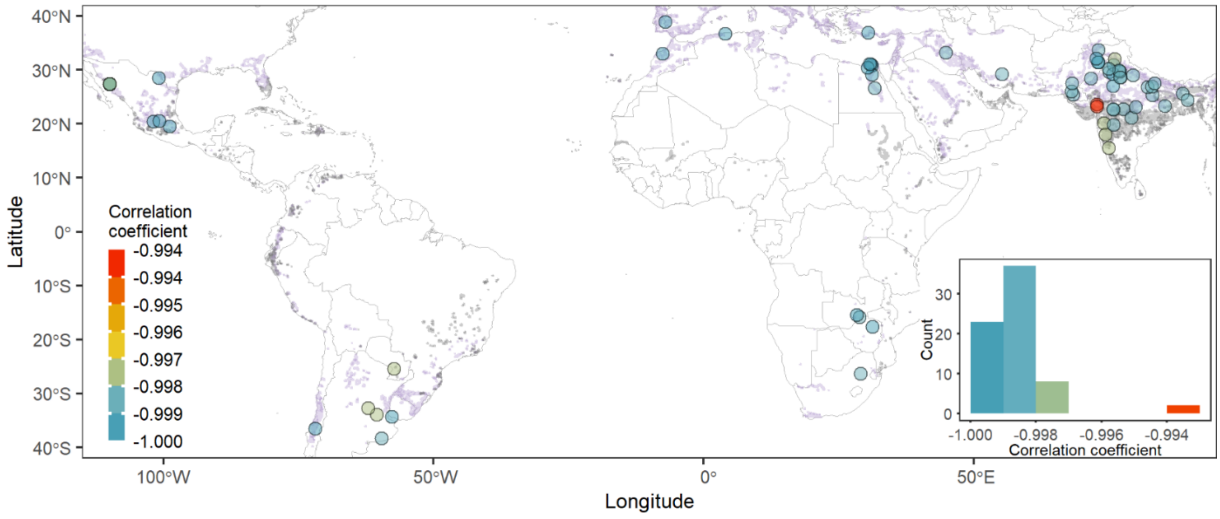

Fig. S13 Spearman correlation between the duration of late reproductive phase and the maximum leaf area index of virtual genotypes of spring wheat with the same duration to anthesis at 70 sites of irrigated mega-environments.
